# 1-*O*-Alkylglycerol Ethers from the Marine Sponge *Guitarra abbotti* and Their Cytotoxic Activity

**DOI:** 10.3390/md20070409

**Published:** 2022-06-22

**Authors:** Sergey A. Dyshlovoy, Sergey N. Fedorov, Vasily I. Svetashev, Tatiana N. Makarieva, Anatoliy I. Kalinovsky, Olga P. Moiseenko, Vladimir B. Krasokhin, Larisa K. Shubina, Alla G. Guzii, Gunhild von Amsberg, Valentin A. Stonik

**Affiliations:** 1 Department of Oncology, Hematology and Bone Marrow Transplantation with Section Pneumology, Hubertus Wald Tumorzentrum—University Cancer Center Hamburg (UCCH), University Medical Center Hamburg-Eppendorf, 20251 Hamburg, Germany; g.von-amsberg@uke.de; 2 Martini-Klinik, Prostate Cancer Center, University Hospital Hamburg-Eppendorf, 20251 Hamburg, Germany; 3 Institute of Science-Intensive Technologies and Advanced Materials, Far Eastern Federal University, 690091 Vladivostok, Russia; 4 G.B. Elyakov Pacific Institute of Bioorganic Chemistry, Far-East Branch of the Russian Academy of Sciences, 690022 Vladivostok, Russia; makarieva@piboc.dvo.ru (T.N.M.); kaaniw@piboc.dvo.ru (A.I.K.); olga-moiseenko@yandex.ru (O.P.M.); shubina@piboc.dvo.ru (L.K.S.); gagry@rambler.ru (A.G.G.); stonik@piboc.dvo.ru (V.A.S.); 5 National Scientific Center of Marine Biology, Far-East Branch of the Russian Academy of Sciences, 690041 Vladivostok, Russia; vsvetashev@mail.ru

**Keywords:** marine sponge, 1-*O*-alkylglycerol ethers, saturated and unsaturated, NMR, GC/MS, diacetates, bismethylsulfides, trimethylsilyl ethers, cytotoxic activity, MAP kinases

## Abstract

The cytotoxicity-bioassay-guided fractionation of the ethanol extract from the marine sponge *Guitarra abbotti*, whose 1-*O*-alkyl-*sn*-glycerol ethers (AGEs) have not been investigated so far, led to the isolation of a complex lipid fraction containing, along with previously known compounds, six new lipids of the AGE type. The composition of the AGE fraction as well as the structures of 6 new and 22 previously known compounds were established using ^1^H and ^13^C NMR, GC/MS, and chemical conversion methods. The new AGEs were identified as: 1-*O*-(Z-docos-15-enyl)-*sn*-glycerol (**1**), 1-*O*-(Z-docos-17-enyl)-*sn*-glycerol (**2**), 1-*O*-(Z-tricos-15-enyl)-*sn*-glycerol (**3**), 1-*O*-(Z-tricos-16-enyl)-*sn*-glycerol (**4**), 1-*O*-(Z-tricos-17-enyl)-*sn*-glycerol (**5**), and 1-*O*-(Z-tetracos-15-enyl)-*sn*-glycerol (**6**). The isolated AGEs show weak cytotoxic activity in THP-1, HL-60, HeLa, DLD-1, SNU C4, SK-MEL-28, and MDA-MB-231 human cancer cells. A further cytotoxicity analysis in JB6 P^+^ Cl41 cells bearing mutated MAP kinase genes revealed that ERK2 and JNK1 play a cytoprotective role in the cellular response to the AGE-induced cytotoxic effects.

## 1. Introduction

It is well known that many marine invertebrates, including sponges [1,2,3,4,5,6,7,8,9,10,11,12,13], corals [14,15,16,17,18,19], mollusks [14,20,21,22,23,24,25], starfish [26], holothurians [27,28], crabs [21], and ascidians [29] as well as some marine algae [30] are well-established sources of a variety of natural 1-*O*-alkyl-*sn*-glycerol ethers (AGEs); for a review, see [31,32]. AGE molecules consist of a long-chain alkyl moiety linked to the glycerol by an ether bond at the *sn*-1 position. Previously, it was established that all the natural AGEs are enantiomerically pure, with an *S* configuration of the asymmetric carbon in the glycerol moiety [33,34]. The AGEs metabolism is controlled by the activity of alkylglycerol monooxygenase (AGMO), which is capable of cleavage of the ether bond of AGEs [35,36]. In marine invertebrates, AGEs mostly present as complex inseparable mixtures of ethers containing different alkyl radicals of various lengths and levels of unsaturation [1,2,3,4,5,6,7,8,9,10,11,12,13,14,15,16,17,18,19,20,21,22,23,24,25,26,27,28,29,30,31,32]. It is highly likely that AGEs are used by invertebrates as a part of their chemical defense against predators. Some reports showed the toxicity of AGEs against fish [10] as well as an antifeedant effect against starfish [14,20]. Both natural and synthetic AGEs possess various biological activities; for a review, see [37]. Among others, there are such useful properties as anticancer [2,3,17,18,21,29,38,39,40,41,42,43,44,45], anti-influenza [46], antibacterial [16,30,47,48], antifungal [49], and antifouling [50] activities found in these metabolites. AGEs have been described to reduce cardiovascular and rheumatoid arthritis risk factors [51], the side effects of radiotherapy [52,53,54], obesity [55,56,57,58], microglial activation, and a neuropathic pain [22,59]. Additionally, AGEs are effective adjuvants [60,61], which modulate endothelial cell permeability [62], the immune response in vitro and in vivo [63,64,65,66], open the blood–brain barrier [67,68], are able to penetrate the skin [69], and improve sperm motility [70]. The search for new structural variants of these lipids and the study of their diversity and biological activities represent an interesting aspect of the marine natural products research field.

**Figure 1:**
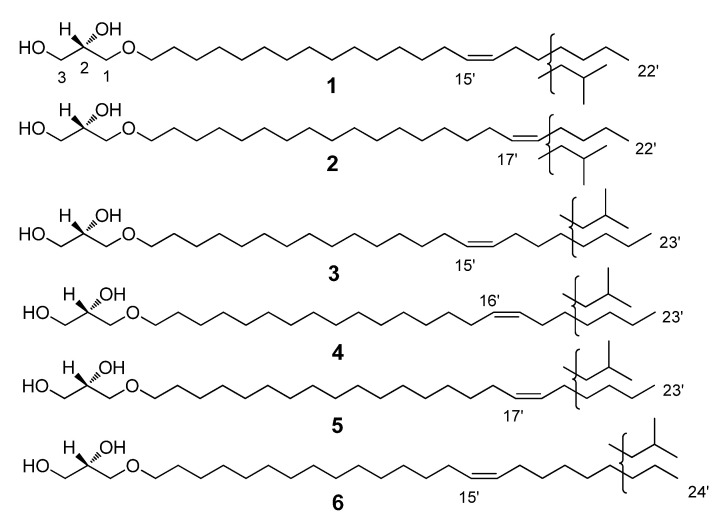
Structures of the new compounds, **1**–**6**: 1-*O*-(Z-docos-15-enyl)-sn-glycerol (**1**), 1-*O*-(Z-docos-17-enyl)-sn-glycerol (**2**), 1-*O*-(Z-tricos-15-enyl)-sn-glycerol (**3**), 1-*O*-(Z-tricos-16-enyl)-sn-glycerol (**4**), 1-*O*-(Z-tricos-17-enyl)-sn-glycerol (**5**), and 1-*O*-(Z-tetracos-15-enyl)-sn-glycerol (**6**).

In a continuation of the studies on cytotoxic marine natural products and their synthetic analogues [71,72,73,74], we have examined the ethanol extract of the cold-water marine sponge *Guitarra abbotti*, which exhibited a cytotoxic activity against human leukemia THP-1 cells in a screening assay. The AGEs of this sponge have never been studied before. A bioassay-guided fractionation of the crude extract led to the isolation of an AGE mixture containing 6 new (**1**–**6**, Figure 1) and 22 known (**7**–**28**, Table 1 and Table 2) compounds of this class. The structures of the AGEs and the composition of the mixture as well as its cytotoxic properties and the partial molecular mechanism of its cytotoxic action are reported.

## 2. Results

### 2.1. Composition of the Isolated AGE Fraction

The isolated AGE mixture exhibited the characteristic signals in the ^1^H and 13C NMR spectra, similar to those previously published [5,6,9,10,12] (see Materials and Methods).

To establish the composition and the structures of the isolated AGEs, including the percentages of the compounds, the AGEs were converted to their corresponding TMS ethers and further analyzed with GC/MS. The GC data and characteristic MS fragmentation of the TMS derivatives of AGEs are shown in Table 1. The data analysis revealed that 22 of the identified AGEs (compounds **7**–**28**) exhibit very similar fragmentations to compounds isolated earlier from other biological sources [1,2,3,4,5,6,7,8,9,10,11,12,13,14,15,16,17,18,19,20,21,22,23,24,25,26,27,28,29,30,31,32]. This fragmentation, in the majority of cases, was related to the loss of a methyl group, trimethylsilanol, (trimethylsilanoxy)methylene, and tetramethylene groups. Nevertheless, six previously unknown compounds (Figure 1) required a more detailed analysis of their structures.

**Figure 2:**
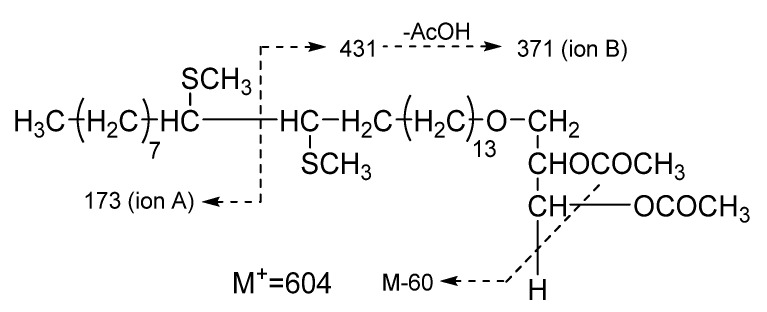
Characteristic MS fragmentation of DMDS derivative of acetylated 1-*O*-(Z-tetracos-15’-enyl)-*sn*-glycerol (**6**).

Next, to determine the exact double bond position in the unsaturated AGEs, correspondent acetates and then their dimethyldisulfide derivatives were synthesized and further investigated with GC/MS. The fragmentation scheme is represented in Figure 2, and the generated GC data and characteristic MS fragmentations are shown in Table 2. The S configuration of the stereogenic center at C-2 of the glycerol moiety of AGEs is the same as was determined for the structural analogues previously isolated from marine invertebrates [1,2,3,4,5,6,7,8,9,10,11,12,13,14,15,16,17,18,19,20,21,22,23,24,25,26,27,28,29,30,31,32]. Thus, 18 unsaturated substances were identified, 6 of which, with carbon chain lengths of C_22_-C_24_, were identified as new compounds: 1-*O*-(Z-docos-15’-enyl)-*sn*-glycerol (**1**); 1-*O*-(Z-docos-17’-enyl)-*sn*-glycerol (**2**); 1-*O*-(Z-tricos-15’-enyl)-*sn*-glycerol (**3**); 1-*O*-(Z-tricos-16’-enyl)-*sn*-glycerol (**4**); 1-*O*-(Z-tricos-17’-enyl)-*sn*-glycerol (**5**); and 1-*O*-(Z-tetracos-15’-enyl)-*sn*-glycerol (**6**) (Figure 1, Table 2).

Apart from the 6 new compounds, **1**–**6**, the 22 previously known AGEs (10 saturated and 12 unsaturated) were identified in the isolated mixture: 1-*O*-(tetradecanyl)-*sn*-glycerol (**7**); 1-*O*-(pentadecanyl)-*sn*-glycerol (T_R_ 16.58 of TMS derivarive (TMS-d), Table 1) (**8**); 1-*O*-(pentadecanyl)-*sn*-glycerol (T_R_ 16.87 of TMS-d, Table 1) (**9**); 1-*O*-(hexadecanyl)-*sn*-glycerol (T_R_ 17.35 of TMS-d, Table 1) (**10**); 1-*O*-(hexadecanyl)-*sn*-glycerol (T_R_ 17.64 of TMS-d, Table 1) (**11**); 1-*O*-(heptadecanyl)-*sn*-glycerol (T_R_ 18.10 of TMS-d, Table 1) (**12**); 1-*O*-(heptadecanyl)-*sn*-glycerol (T_R_ 18.18 of TMS-d, Table 1) (**13**); 1-*O*-(heptadecanyl)-*sn*-glycerol (T_R_ 18.38 of TMS-d, Table 1) (**14**); 1-*O*-(octadecanyl)-*sn*-glycerol (T_R_ 18.80 of TMS-d, Table 1) (**15**); 1-*O*-(octadecanyl)-*sn*-glycerol (T_R_ 19.10 of TMS-d, Table 1) (**16**); 1-*O*-(Z-hexadec-7’-enyl)-*sn*-glycerol (T_R_ 44.69 of DMDS-d, Table 2) (**17**); 1-*O*-(Z-hexadec-9’-enyl)-*sn*-glycerol (T_R_ 45.00 of DMDS-d, Table 2) (**18**); 1-*O*-(Z-hexadec-11’-enyl)-*sn*-glycerol (T_R_ 45.69 of DMDS-d, Table 2) (**19**); 1-*O*-(Z-hexadec-13’-enyl)-*sn*-glycerol (T_R_ 47.69 of DMDS-d, Table 2) (**20**); 1-*O*-(Z-octadec-9-enyl)-*sn*-glycerol (T_R_ 51.10 of DMDS-d, Table 2) (**21**); 1-*O*-(Z-octadec-11-enyl)-*sn*-glycerol (T_R_ 51.40 of DMDS-d, Table 2) (**22**); 1-*O*-(Z-octadec-12-enyl)-*sn*-glycerol (T_R_ 51.62 of DMDS-d, Table 2) (**23**); 1-*O*-(Z-octadec-13-enyl)-*sn*-glycerol (T_R_ 52.13 of DMDS-d, Table 2) (**24**); 1-*O*-(Z-cos-11’-enyl)-*sn*-glycerol (T_R_ 58.48 of DMDS-d, Table 2) (**25**); 1-*O*-(Z-cos-13’-enyl)-*sn*-glycerol (T_R_ 59.39 of DMDS-d, Table 2) (**26**); 1-*O*-(Z-cos-15’-enyl)-*sn*-glycerol (T_R_ 60.29 of DMDS-d, Table 2) (**27**); and 1-*O*-(Z-tetracos-17’-enyl)-*sn*-glycerol (T_R_ 87.30 of DMDS-d, Table 2) (**28**) [1,2,3,4,5,6,7,8,9,10,11,12,13,14,15,16,17,18,19,20,21,22,23,24,25,26,27,28,29,30,31,32].

### 2.2. Anticancer Effects of the Isolated AGE Fraction

The cytotoxicity of the isolated AGE mixture was evaluated using the MTS viability assay in seven human cancer cell lines [75]. The calculated IC_50_ values are shown in Table 3.

**Figure 3:**
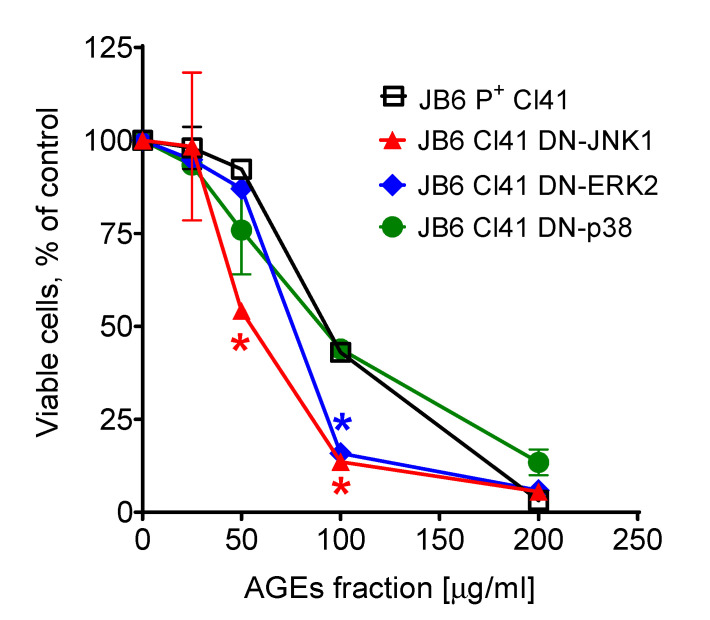
Cytotoxic effect of the isolated AGE mixture in JB6 P^+^ Cl41 cells and its stable transfectants, JB6 Cl41 DN-JNK1, JB6 Cl41 DN-p38, and JB6 Cl41 DN-ERK2 cells. * Significant different (*p* < 0.05, Student’s t-test) in the viability of the cells bearing the mutant kinase compared to the viability measured in JB6 P^+^ Cl41 cells (wild-type) exposed to the same concentration of the AGE mixture.

We used dominant negative mutant (DNM) JB6 Cl41 cells to elucidate the roles of the three major MAP kinases (MAPKs) in the cytotoxic effects of the isolated AGE mixture. The cytotoxic activity of the isolated AGE fraction against the normal mouse epidermal cell line JB6 Cl41 and its stable transfectants JB6 Cl41 DN-JNK1, JB6 Cl41 DN-p38, and JB6 Cl41 DN-ERK2 cells is shown in Figure 3. The DN cell lines contain a mutation in the kinase coding gene, which leads to the inactivation of the corresponding kinase in the cells. An examination of the drug effect in the cells bearing inactivated kinase may help to reveal a role of the kinase in the therapeutic effect of the drug. Our experiments indicated that an inactivation of ERK2 and JNK1 results in higher cytotoxicity of the tested AGE fraction. These data suggest that ERK2 and JNK1 (but not p38) play a cytoprotective role in the cellular response to the AGE treatment.

## 3. Discussion

The mixture of the native AGEs was isolated from the extracts of the sponge *G. abbotti* without any prior derivatization or hydrolysis. A comparison with the literature data [5,6,9,10,12] for the ^1^H NMR spectrum of the AGE mixture (see Materials and Methods) made it possible to correlate the signals at δ 3.55–3.86 ppm with the protons of the glyceride group, those at δ 3.46 ppm with the protons at C1’ of the alkyl chains, those at δ 1.57 ppm with the protons at C2’ of the alkyl chains, those at δ 1.22–1.38 ppm with the protons of other CH_2_ groups in the alkyl chains, those at δ 5.34 ppm with the protons at double bonds, those at δ 2.01 ppm with the allylic protons, and those at 0.85–0.88 ppm with protons of the terminal methyl groups of the alkyl chain. Thus, the analysis of the NMR spectra showed that the mixture consists of saturated and unsaturated AGEs, the side chains of which may contain *n*- and *iso*- terminal methyl groups (Figures S1–S3, Supplementary Materials). The Z-geometry of the double bonds in the unsaturated lipids was determined by the small coupling constant, J = 4.6 Hz, of the olefinic proton signal in the ^1^H spectrum (according to 4.5–6.3 Hz for the Z-configuration in [6,9]) as well as by the shielded chemical shifts of the allylic (δ 26.1 ppm) and olefinic (δ 129.9 ppm) carbon atoms in the ^13^C NMR spectrum of the AGE mixture [6,76]. The *S* configuration at the asymmetric carbon of the glycerol moiety was previously established for all the natural AGEs [1,2,3,4,5,6,7,8,9,10,11,12,13,14,15,16,17,18,19,20,21,22,23,24,25,26,27,28,29,30,31,32]. Therefore, we assume the same configuration for our isolates. To establish the chemical structures of the lipids of the isolated AGE fraction, first their TMS ethers were obtained and analyzed by the GC/MS method (Figures S4 and S5). The GC data and characteristic MS fragmentation of the TMS derivatives of AGEs (see Table 1) revealed the structures of the 10 previously known saturated AGEs that are part of the isolated alkylglycerol mixture [1,2,3,4,5,6,7,8,9,10,11,12,13,14,15,16,17,18,19,20,21,22,23,24,25,26,27,28,29,30,31,32]. The GC/MS data not only confirmed the presence of both saturated and unsaturated AGEs in the mixture but also indicate their approximately equal ratio. Thus, the two main saturated AGEs, containing side chains with 16 and 17 carbon atoms, accounted for 21% and 13% of the total mixture, while the two main unsaturated AGEs with 22 and 24 carbon atoms in the side chains accounted for 16% and 18%, respectively (Table 1). The positions of the double bonds in the unsaturated AGEs were determined based on the analysis of the characteristic MS fragmentation of the DMDS derivatives of the acetylated AGEs (Table 2, Figure 2, Figures S6 and S7). As a result, after analyzing the mass spectra of the TMS and DMDS derivatives of the unsaturated lipids that are part of the AGE mixture, 18 unsaturated substances were structurally identified, 6 of which, with carbon chain lengths of C_22_-C_24_, are new compounds. Their chemical structures were established as follows: 1-*O*-(Z-docos-15’-enyl)-*sn*-glycerol (**1**), 1-*O*-(Z-docos-17’-enyl)-*sn*-glycerol (**2**), 1-*O*-(Z-tricos-15’-enyl)-*sn*-glycerol (**3**), 1-*O*-(Z-tricos-16’-enyl)-*sn*-glycerol (**4**), 1-*O*-(Z-tricos-17’-enyl)-*sn*-glycerol (**5**), and 1-*O*-(Z-tetracos-15’-enyl)-*sn*-glycerol (**6**) (Figure 1).

Different studies suggest that in a living organism AGEs serve as precursors and can be enzymatically converted into various types of biologically active ether lipid compounds [77]. At the same time, enzymatically unmodified AGEs also exhibit biological activities. Ether lipids execute various biological functions, including the modulation of several important signaling pathways [78,79]. Thus, AGEs can be used to generate plasmalogens or platelet-activating factor (PAF) in various biological systems in vitro and in vivo [80,81]. Plasmalogens are important membrane constituents as well as regulators of cholesterol biosynthesis and transport; they play a role in intercellular communication, cell migration, and signal transduction [77,80]. PAF, in turn, is involved in a wide range of membrane-dependent processes and has potent biological activities towards various cell types and systems of an organism, including inflammation, circulation, reproduction, and development; for a review, see [82,83]. A deficiency of plasmalogens can impair some membrane-associated signaling, such as AKT/PKB, leading to a myelination defect [84], while supplementation with plasmalogens modulates important signaling pathways associated with ERK, AKT, p38, and JNK [85,86,87]. On the other hand, there are some studies which highlight the bioactivity of non-modified AGEs. For example, AGEs were found to accumulate in adipocytes upon differentiation and regulate adipogenesis [58], induce calcium influx in human lymphocytes [88], and inhibit PKC in vitro and in vivo [89].

MAP kinases play roles in various biological processes [85,86,87]. Due to the broad spectrum of biological targets and processes affected by AGEs, we have evaluated the importance of MAPK-dependent pathways for the cytotoxic effect of the isolated AGE fraction. In our study, using mouse epidermal JB6 Cl41 cells we demonstrated that ERK2- and JNK1- (but not p38) related pathways may play a cytoprotective role in the cytotoxic response to AGE exposure (Figure 3). Thus, the cells bearing knocked-out ERK2 and JNK1 genes are more sensitive to the cytotoxic effect of the isolated AGE fraction compared to the original JB6 Cl41 cells expressing nonmutant MAPK genes. Further experiments, including those examining an effect on MAPK activation, would be necessary to validate this speculation. Within the current study, the further examination of biological activity could not be performed due to the limited amount of the isolated AGE fraction.

The inhibitory effect of AGEs on protein kinase C [89] suggests their potent action against proliferative diseases. Indeed, it was shown that the treatment with AGEs prevents tumor growth in vivo due to the inhibition of angiogenesis [38,39,41,44]. Interestingly, unsaturated AGEs in some assays are significantly more active when compared to saturated molecules [38,39].

We demonstrated here that the AGE mixture inhibits the viability of seven human cancer cell lines representing different cancer entities (Table 3). In all the cell lines, apart from HL-60 and SK-MEL-28, the cytotoxic effects of AGEs from marine invertebrates were reported for the very first time. The highest cytotoxic activity, with IC_50_ = 35.9 µg/mL (Table 3), was shown for THP-1 cells, highlighting the potential of these and related compounds for the treatment of human leukemia. However, it should be noted that the cytotoxic activity of the isolated AGE fraction was overall rather weak.

## 4. Materials and Methods

### 4.1. General Procedures

The ^1^H and ^13^C NMR spectra were recorded on a Bruker DRX-300 spectrometer (Bruker GmbH, Bremen, Germany) at 300 and 75 MHz, respectively, in CDCl_3_ with tetramethylsilane as an internal standard. 

The GC/MS data for the TMS derivatives were obtained using a Hewlett Packard GC HP6890 instrument (Agilent Technologies Inc., Santa Clara, CA, USA) with an HP5973 mass-selective detector. The injector and transfer line temperatures were 270 °C. A Hewlett Packard HP-5MS capillary column (Agilent Technologies Inc., Santa Clara, CA, USA), 30 m × 0.25 mm, phase layer 0.25 µm, was used at 100 °C with a 2 °C/min ramp to 270 °C, which was held for 30 min. The column contained 5% phenylmethylsiloxane and it was used as the mobile phases at a flow rate of 1 mL/min. The sample was dissolved in chloroform at a concentration of 10 mg/mL. The injection volume was 0.2 μL, and the split ratio was 15:1. The mass spectra were recorded at 70 eV.

The GC/MS data for the DMDS-derived AGEs were obtained using a Shimadzu GCMS-QP5050 instrument (Shimadzu, Kyoto, Japan). An MDN-5S capillary column (Shimadzu, Kyoto, Japan), 30 m × 0.25 mm, phase layer 0.25 µm, was used at 200 °C with a 2 °C/min ramp to 300 °C, which was held for 45 min. The split ratio was 15:1, and the flow rate was 1 mL/min. The injector temperature was 270 °C. The mass spectra were recorded at 70 eV.

Low-pressure column liquid chromatography was performed using KSK silica gel (50–100 μm, Sorbpolymer, Krasnodar, Russia). Sorbfil silica gel plates (4.5 × 6.0 cm, 5–17 μm, Sorbpolymer, Krasnodar, Russia) were used for TLC. HPLC was performed using an Agilent 1100 instrument (Agilent Technologies Inc, Santa Clara, CA, USA) equipped with a differential refractometer on a Diasorb-60-Silicagel (250 × 4.6 mm) column (BioChemMak, Moscow, Russia). Cells were counted using an Olympus inverted research microscope (Olympus, Tokyo, Japan). The absorption of MTS/farmazan was measured spectrophotometrically using the μQuant microplate reader (Bio-Tek Instruments Inc., Winooski, VT, USA).

### 4.2. Reagents

Minimum essential medium (MEM), DMEM, and RPMI medium were purchased from BioloT (Sankt-Peterburg, Russian Federation); fetal bovine serum (FBS) was purchased from Thermo Fisher Scientific (Cramlington, Northumberland, UK); penicillin/streptomycin was purchased from Bio-Whittaker (Walkersville, MD, USA); and L-glutamine was purchased from Mediatech Inc. (Herndon, VA, USA). The Cell Titer 96 Aqueous One Solution Reagent (MTS) kit for the cell viability assay was purchased from Promega (Madison, WI, USA).

### 4.3. Animal Material

The marine sponge *Guitarra abbotti* (family Guitarridae, order Poecilosclerida) was collected by dredging at the depth of 109 m at 48°00′08′′ N, 153°20′07′′ E, Kuril Islands, the Sea of Okhotsk, Pacific Ocean. A voucher specimen is kept in the collection of the G.B. Elyakov Pacific Institute of Bioorganic Chemistry. The taxonomic identification was performed by V.B. Krasokhin.

### 4.4. Extraction and Isolation

Animal materials (1050 g, wet weight) were extracted with EtOH (2 L) immediately after collection. After evaporation in vacuo, the ethanol extract was re-dissolved in 200 mL of EtOH/H_2_O (5:1, *v*/*v*) and extracted with 3 × 200 mL of *n*-hexane. The aqueous-ethanolic and combined *n*-hexane fractions after evaporation were subjected to an evaluation of cytotoxic activity against human leukemia THP-1 cells by the MTS method and showed IC_50_ = 0.6 and 0.5 mg/mL, respectively. The *n*-hexane fraction was selected for the further isolation of anticancer compounds. This fraction (3.525 g) was evaporated and subjected to column chromatography on a silica gel column (diameter/length = 6:14 cm) using an *n*-hexane/AcOEt gradient as an eluent with *n*-hexane to AcOEt ratios of 19:1, 9:1, 4:1, 3:2, 1:1, 2:3, 1:4, 1:9, and 1:19 (*v*/*v*), then with 100% EtOAc and then with 100% EtOH. A 200 mL volume of eluent was used for the elution of each fraction. A subsequent evaluation of the cytotoxic activity revealed that the fraction eluted with *n*-hexane/AcOEt (2:3, *v*/*v*) possessed the highest cytotoxicity towards THP-1 cells, IC_50_ = 125 μg/mL. This fraction (299 mg) was further subjected to the same silica gel column chromatography using 330 mL of *n*-hexane/AcOEt (3:2, *v*/*v*) and 400 mL of *n*-hexane/AcOEt (1:1, *v*/*v*) as eluents. The collected fractions (10 mL each) were examined using TLC (SiO_2_ and *n*-hexane/AcOEt (1:1, *v*/*v*)) as a chromatographic system, with the further detection of spots on the TLC chromatograms using H_2_SO_4_/EtOH (1:9, *v*/*v*). Following the TLC analysis, three fractions were obtained, i.e., (i) fraction #1 (one grey spot on the TLC, R_f_ = 0.5); (ii) fraction #2 (one violet spot on the TLC with R_f_ = 0.2); and (iii) fraction #3 (two spots on the TLC, R_f_ = 0.5 and R_f_ = 0.2). The examination of the cytotoxic activity against human THP-1 cells revealed fraction #2 to be the most active, with IC_50_ = 62.5 μg/mL. Further, fraction #2 (185 mg, dry weight) was additionally purified by HPLC using a Diasorb-60-Silica gel column and *n*-hexane/AcOEt (3:2, *v*/*v*) as the eluent. As a result, the more active mixture (IC_50_ = 35.9 μg/mL in THP-1 cells) of the purified 1-*O*-alkylglycerol ethers (AGEs) was obtained (160 mg, dry weight).

### 4.5. Characterization of the Purified 1-O-Alkylglycerol Ether (AGE) Mixture

The chemical nature of the isolated purified fraction was established using their NMR spectra. Generally, the next spectroscopic information was obtained. 

^1^H NMR (CDCl_3_, 300 MHz) δ 0.85 d, J = 6.6 Hz, *iso*-CH_3_; 0.88 t, *J* = 6.7 Hz, *n*-CH_3_; 1.22-1.38, m, (CH_2_)_n_, aliphatic chain; 1.57, quint, *J* = 6.9 Hz, 2H-2’; 2.01, q, *J* = 6.3 Hz, 2H allylic protons; 3.46, td, *J* = 6.6 Hz, *J* = 1.6 Hz, 2H-1’; 3.55, dd, *J* = 9.2 Hz, *J* = 3.5 Hz, 2H-1; 3.64 dd, *J* = 11.4 Hz, *J* = 5.2 Hz, 1H-3a; 3.72 dd, *J* = 11.4 Hz, *J* = 3.8 Hz, 1H-3b; 3.86, m, 1H-2; 5.34, t, *J* = 4.6 Hz, 2H, olefinic protons.

^13^C NMR (CDCl_3_, 75 MHz) δ 14.10 (**C**H_3_); 22.64 (**C**H_2_); 26.05 (2**C**H_2_, allylic carbons); 27.19 (**C**H_2_); 28.97 (**C**H_2_); 29.07 (**C**H_2_); 29.30 (**C**H_2_); 29.34 (**C**H_2_); 29.44 (**C**H_2_); 29.54 (**C**H_2_); 29.66 (**C**H_2_); 31.76 (**C**H_2_); 31.90 (**C**H_2_); 64.24 (**C**H_2_-3); 70.40 (**C**H-2); 71.84 (**C**H_2_-1); 72.48 (**C**H_2_-1’); 129.88 (**C**H=**C**H).

These data indicate the AGE nature of the compounds in the isolated mixture. These compounds contain a glycerol moiety linked by an ether bond with a fatty alcohol residue. In the analyzed compounds, the fatty alcohol residues contain either normal or iso-ends, and some of them have an additional disubstituted double bond. The majority of the identified metabolite compounds have previously been isolated from different biological sources. However, six of them (**1**–**6**) were new natural products (see below).

### 4.6. Preparation of Trimethylsilyl Derivatives (TMS-d) and Their GC/MS Analysis

First, 0.1 mg of dried AGE mixture was treated with 0.1 mL of BSTFA (Supelco, Bellefonte, PA, USA) at 60 °C for 1 h to convert the AGEs to their trimethylsilyl derivatives (TMS-d). The obtained TMS-d were analyzed by the GC/MS method (Figures S4 and S5, Supplementary Materials). The mass spectra of TMS-d of the new and previously known AGEs, although the exact position of the double bond could not be precisely determined in some cases, provide valuable information about their structures.

The mass spectra data of the TMS-d for the previously known compounds (**7**–**28**) are given below. 

TMS-d of 1-*O*-(tetradecanyl)-*sn*-glycerol (**7**), T_R_ 16.07, m/z 417, 285, 205, 147, 117, 103, 73; 57; TMS-d of 1-*O*-(pentadecanyl)-*sn*-glycerol (**8**), T_R_ 16.58, m/z 431, 356, 299, 205, 147, 117, 103, 73; 57; TMS-d of 1-*O*-(pentadecanyl)-*sn*-glycerol (**9**), T_R_ 16.87, m/z 431, 356, 299, 205, 147, 117, 103, 73; 57; TMS-d of 1-*O*-(hexadecanyl)-*sn*-glycerol (**10**), T_R_ 17.35, m/z 445, 370, 313, 205, 147, 117, 103, 73, 57; TMS-d of 1-*O*-(Z-hexadec-7’ or 9’ or 11’ or 13’-enyl)-*sn*-glycerol (**17** or **18** or **19** or **20)**, T_R_ 17.51, m/z 458, 443, 355, 311, 265, 205, 147, 117, 103, 73, 55; TMS-d of 1-*O*-(Z-hexadec-7’ or 9’ or 11’ or 13’-enyl)-*sn*-glycerol (**17** or **18** or **19** or **20)**, T_R_ 17.59, m/z 458, 355, 311, 265, 205, 147, 117, 103, 73, 55; TMS-d of 1-*O*-(hexadecanyl)-*sn*-glycerol (**11**), T_R_ 17.64, m/z 445, 370, 313, 205, 147, 117, 103, 73, 57; TMS-d of 1-*O*-(heptadecanyl)-*sn*-glycerol (**12**), T_R_ 18.10, m/z 459, 384, 327, 205, 147, 117, 103, 73, 57; TMS-d of 1-*O*-(heptadecanyl)-*sn*-glycerol (**13**), T_R_ 18.18, m/z 459, 384, 327, 205, 147, 117, 103, 73, 57; TMS-d of 1-*O*-(heptadecanyl)-*sn*-glycerol (**14**), T_R_ 18.38, m/z 459, 384, 327, 205, 147, 117, 103, 73, 57; TMS-d of 1-*O*-(octadecanyl)-*sn*-glycerol (**15**), T_R_ 18.80, m/z 473, 398, 341, 205, 147, 117, 103, 73, 57; TMS-d of 1-*O*-(Z-octadec-9’ or 11’ or 12’ or 13’-enyl)-*sn*-glycerol (**21** or **22 or 23** or **24)**, T_R_ 18.93, m/z 486, 471, 293, 205, 147, 117, 103, 73, 55; TMS-d of 1-*O*-(Z-octadec-9’ or 11’ or 12’ or 13’-enyl)-*sn*-glycerol (**21** or **22** or **23** or **24)**, T_R_ 18.99, m/z 486, 471, 293, 205, 147, 117, 103, 73, 55.; TMS-d of 1-*O*-(octadecanyl)-*sn*-glycerol (**16**), T_R_ 19.10, m/z 473, 398, 341, 205, 147, 117, 103, 73, 57; TMS-d of 1-*O*-(Z-cos-11’ or 13’ or 15’-enyl)-*sn*-glycerol (**25** or **26** or **27)**, T_R_ 20.38, m/z 514, 321, 205, 147, 117, 103, 73, 55; TMS-d of 1-*O*-(Z-tetracos-17’-enyl)-*sn*-glycerol (**28**), T_R_ 23.43, m/z 480, 467, 390, 377, 205, 147, 117, 103, 73, 55.

For the six new identified compounds (**1**–**6**) (Figure 1), the mass spectra of their TMS-d are given below.

TMS-d of 1-*O*-(Z-docos-15’-enyl)-*sn*-glycerol (**1**), retention time (T_R_) 21.74, m/z 542, 527, 395, 349, 205, 147, 117, 103, 73, 55; TMS-d of 1-*O*-(Z-docos-17’-enyl)-*sn*-glycerol (**2**), T_R_ 21.81, m/z 542, 527, 395, 349, 205, 147, 117, 103, 73, 55; TMS-d of 1-*O*-(Z-tricos-15’-enyl)-*sn*-glycerol (**3**) or 1-*O*-(Z-tricos-16’-enyl)-*sn*-glycerol (**4**), or 1-*O*-(Z-tricos-17’-enyl)-*sn*-glycerol (**5**), T_R_ 22.56, m/z 363, 205, 147, 117, 103, 73, 55; TMS-d of 1-*O*-(Z-tetracos-15’-enyl)-*sn*-glycerol (**6**), T_R_ 23.35, m/z 480, 467, 390, 377, 205, 147, 117, 103, 73, 55.

### 4.7. Preparation of Acetate Derivatives

First, 1.3 mg of the dried AGE mixture was treated with 0.4 mL of Ac_2_O/Py (1:1, *v*/*v*) at RT overnight to convert the AGEs to their diacetate derivatives. Then, 2 mL of EtOH was added, and the sample was dried in vacuo.

### 4.8. Preparation of Dimethyldisulfide Acetate Derivatives (DMDS-d) of AGEs and Their GC/MS Analysis

First, 1.2 mg of dried AGE acetate mixture (see above) was mixed with 0.2 mL of dimethyldisulfide (DMDS) and 0.05 mL of iodine solution in Et_2_O (60 mg/mL) and incubated at RT overnight. Then, 5 mL of *n*-hexane was added, and the mixture was washed with 5 mL of an aqueous 5% solution of Na_2_S_2_O_3_ × 5H_2_O until the color of iodine disappeared. The *n*-hexane fraction was separated, and the reaction products were extracted one more time from the polar fraction using *n*-hexane (1 mL). The combined *n*-hexane fractions were dried over sodium sulfate, evaporated in vacuo, and re-dissolved in hexane for further analysis.

Mass spectra of the DMDS-d of the new AGEs, **1**–**6**:

DMDS-d of 1-*O*-(Z-docos-15’-enyl)-*sn*-glycerol (**1**), T_R_ 70.67, m/z 576, 529, 431, 371, 311, 285, 263, 255, 206, 159, 145, 97, 83, 69, 55; DMDS-d of 1-*O*-(Z-docos-17’-enyl)-*sn*-glycerol (**2**), T_R_ 72.07, m/z 576, 529, 459, 399, 339, 313, 291, 283, 234, 159, 117, 97, 83, 69, 55; DMDS-d of 1-*O*-(Z-tricos-15’-enyl)-*sn*-glycerol (**3**), T_R_ 77.50, m/z 590, 436, 371, 341, 311, 173, 159, 145, 117, 97, 83, 69, 55; DMDS-d of 1-*O*-(Z-tricos-16’-enyl)-*sn*-glycerol (**4**), T_R_ 77.90, m/z 590, 436, 385, 341, 297, 159, 145, 117, 97, 83, 69, 55; DMDS-d of 1-*O*-(Z-tricos-17’-enyl)-*sn*-glycerol (**5**), T_R_ 78.67, m/z 590, 436, 399, 341, 283, 159, 145, 131, 117, 97, 83, 69, 55; DMDS-d of 1-*O*-(Z-tetracos-15’-enyl)-*sn*-glycerol (**6**), T_R_ 86.27, m/z 604, 450, 371, 311, 255, 207, 173, 159, 109, 97, 83, 69, 55.

Mass spectra of the DMDS-d of the previously known AGEs:

DMDS-d of 1-*O*-(Z-hexadec-7’-enyl)-*sn*-glycerol (**17**), T_R_ 44.69, m/z 492, 319, 259, 173, 159, 143, 95, 89, 81, 69, 61, 55; DMDS-d of 1-*O*-(Z-hexadec-9’-enyl)-*sn*-glycerol (**18**), T_R_ 45.00, m/z 492, 347, 287, 171, 159, 145, 95, 89, 81, 69, 61, 55; DMDS-d of 1-*O*-(Z-hexadec-11’-enyl)-*sn*-glycerol (**19**), T_R_ 45.69, m/z 492, 375, 315, 199, 159, 117, 95, 89, 81, 69, 61, 55; DMDS-d of 1-*O*-(Z-hexadec-13’-enyl)-*sn*-glycerol (**20**), T_R_ 47.69, m/z 492, 403, 343, 227, 159, 95, 89, 81, 69, 61, 55; DMDS-d of 1-*O*-(Z-octadec-9’-enyl)-*sn*-glycerol (**21**), T_R_ 51.10, m/z 520, 347, 287, 227, 173, 159, 95, 81, 69, 55; DMDS-d of 1-*O*-(Z-octadec-11’-enyl)-*sn*-glycerol (**22**), T_R_ 51.40, m/z 520, 375, 315, 255, 145, 159, 95, 81, 69, 55; DMDS-d of 1-*O*-(Z-octadec-12’-enyl)-*sn*-glycerol (**23**), T_R_ 51.62, m/z 520, 389, 329, 269, 131, 159, 95, 81, 69, 55; DMDS-d of 1-*O*-(Z-octadec-13’-enyl)-*sn*-glycerol (**24**), T_R_ 52.13, m/z 520, 403, 343, 283, 117, 159, 95, 81, 69, 55; DMDS-d of 1-*O*-(Z-cos-11’-enyl)-*sn*-glycerol (**25**), T_R_ 58.48, m/z 548, 375, 315, 255, 199, 159, 117, 109, 95, 83, 69, 55; DMDS-d of 1-*O*-(Z-cos-13’-enyl)-*sn*-glycerol (**26**), T_R_ 59.39, m/z 548, 403, 343, 227, 159, 117, 109, 95, 83, 69, 55; DMDS-d of 1-*O*-(Z-cos-15’-enyl)-*sn*-glycerol (**27**), T_R_ 60.29, m/z 548, 431, 371, 255, 199, 159, 117, 109, 95, 83, 69, 55; DMDS-d of 1-*O*-(Z-tetracos-17’-enyl)-*sn*-glycerol (**28**), T_R_ 87.30, m/z 604, 459, 399, 339, 283, 159, 145, 97, 83, 69, 55.

### 4.9. Cell Culture

The JB6 P^+^ Cl41 mouse epidermal cell line and its stable transfectants JB6 Cl 41 DN-JNK1, JB6 Cl 41 DN-p38, and JB6 Cl 41 DN-ERK2, which have the knockout JNK1 p38 and ERK2 genes, respectively, were cultured as monolayers at 37 °C and 5% CO_2_ in MEM containing 5% fetal bovine serum (FBS), 2 mM L-glutamine, 100 units/mL penicillin, and 100 mg/mL streptomycin. The human cancer cell lines HL-60 (promyelocytic leukemia), THP-1 (monocytic leukemia), HeLa (cervix carcinoma), SNU C4 (colon cancer), DLD-1 (colon cancer), MDA-MB-231 (breast adenocarcinoma), and SK-MEL-28 (melanoma) were obtained from the American Type Culture Collection (Rockville, MD, USA). The HL-60, THP-1, HeLa, DLD-1, SNU C4, and SK-MEL-28 cancer cell lines were cultured at 37 °C and 5% CO_2_ in RPMI medium containing 10% FBS, 2 mM L-glutamine, 100 units/mL penicillin, and 100 mg/mL streptomycin. The MDA-MB-231 cancer cell line was cultured at 37 °C and 5% CO_2_ in DMEM containing 10% FBS, 2mM L-glutamine, 100 units/mL penicillin, and 100 mg/mL streptomycin. HL-60 and THP-1 cells were cultured as suspensions, and the other cell lines were cultured as monolayers. Information regarding the genetic background of these cell lines is available online at the ATCC website.

### 4.10. Cell Viability Test

The effect of the obtained AGE mixture on the viability of the THP-1, HL-60, HeLa, DLD-1, SNU C4, SK-MEL-28, MDA-MB-231, JB6 P^+^ Cl41, JB6 Cl 41 DN-JNK1, JB6 Cl 41 DN-p38, and JB6 Cl 41 DN-ERK2 cell lines was evaluated using the MTS assay [75]. Briefly, corresponding cells were seeded in 96-well plates (6000 cells per well) and incubated overnight in 100 μL of medium per well for adherent cells, or 50 μL/well for non-adherent cells (THP-1, HL-60). For adherent cells, the media were then replaced with fresh media containing the AGE mixture at various concentrations in a total volume of 0.1 mL per well, and the cells were incubated for 22 h. For suspension cells, 50 μL /well of fresh medium containing the AGE mixture was added, and the cells were incubated for 22 h. Then, 10 mL of the MTS reagent was added into each well, and the MTS reduction was measured spectrophotometrically 2 h later at 492 nm and 690 nm (background) using the µQuant microplate reader.

### 4.11. Statistical Analysis

The statistical analyses were performed using Statistica 6.0 (StatSoft, Inc., Tulsa, OK, USA) or GraphPad Prism v.9.1.1 software (GraphPad Software, San Diego, CA, USA). The results of two independent experiments, each performed in triplicate, were used for the analyses. Significant differences from control were calculated using Student’s t-test. The method of regressions was used to calculate the IC_50_ values.

## 5. Conclusions

In conclusion, 6 new and 22 previously known 1-*O*-alkylglycerol ethers were identified in the AGE fraction isolated from the marine sponge *Guitarra abbotti*. Their structures were established using ^1^H and ^13^C NMR spectroscopy and a GC/MS analysis of their TMS and DMDS derivatives as well as a comparison with the literature data. The isolated AGE fraction consisted of both saturated and unsaturated AGEs, which presented at a nearly equimolar ratio. The isolated AGEs exhibited a rather weak cytotoxic activity towards seven human cancer cell lines. Moreover, the active MAP kinases ERK2 and JNK1 were shown to play a cytoprotective role in the cellular response to the AGE-induced cytotoxic effects.

## Figures and Tables

**Table 1:** GC data and characteristic MS fragmentation of TMS derivatives of AGEs.

# of Compound	Retention Time	Fatty Alcohol Residue	Peak Area, % of Total	Characteristic Ion Fragment				
				M^+^–CH_3_	M^+^–HOSi(CH_3_)_3_–CH_2_OSi(CH_3_)_3_	M^+^–HOSi(CH_3_)_3_	M^+^–HOSi(CH_3_)_3_–C_4_H_9_	M^+^
	13.06	N/I*	0.647					
	13.78	N/I*	0.890					
**7**	16.07	14:0	0.436	417	-	-	285	-
**8** (*iso*)	16.58	15:0	0.404	431	-	356	299	-
**9** (*n*)	16.87	15:0	1.113	431	-	356	299	-
**10** (*iso*)	17.35	16:0	2.514	445	-	370	313	-
**17** or **18** or **19** or **20**	17.51	16n:1	1.973	443	265	-	311	458
**17** or **18** or **19** or **20**	17.59	16n:1	1.168	-	265	-	311	458
**11** (*n*)	17.64	16:0	21.116	445		370	313	-
**12** (*iso*)	18.10	17:0	13.648	459		384	327	-
**13** (*anteiso*)	18.18	17:0	2.181	459		384	327	-
**14** (*n*)	18.38	17:0	1.414	459		384	327	-
**15** (*iso*)	18.80	18:0	0.700	473		398	341	-
**21** or **22** or **23** or **24**	18.93	18n:1	1.243	471	293	396	339	486
**21** or **22** or **23** or **24**	18.99	18n:1	2.118	471	293	396	339	486
**16** (*n*)	19.10	18:0	5.403	473	-	398	341	-
**25** or **26** or **27**	20.38	20n:1	0.400	-	321	-	-	514
**1**	21.74	22n:1	16.036	527	349	-	395	542
**2**	21.81	22n:1	2.860	527	349	-	395	542
**3** or **4** or **5**	22.56	23n:1	0.731	-	363	-	-	-
	23.13	N/I*	1.496					
**6**	23.35	24n:1	1.722	-	377	480	-	-
**28**	23.43	24n:1	18.241	-	377	480	423	-
	25.35	N/I*	1.548					

*—not identified.

**Table 2:** GC data and characteristic MS fragmentation of DMDS derivatives of acetylated AGEs.

# of Compound	Retention Time	Fatty Alcohol Residue	Ion Fragment		
			Ion A	Ion B	M^+^
**17**	44.69	16:1n-7	173	259	492
**18**	45.00	16:1n-9	145	287	492
**19**	45.69	16:1n-11	117	315	492
**20**	47.69	16:1n-13	89	343	492
**21**	51.10	18:1n-9	173	287	520
**22**	51.40	18:1n-11	145	315	520
**23**	51.62	18:1n-12	131	329	520
**24**	52.13	18:1n-13	117	343	520
**25**	58.48	20:1n-11	173	315	548
**26**	59.39	20:1n-13	145	343	548
**27**	60.29	20:1n-15	117	371	548
**1**	70.67	22:1n-15	145	371	576
**2**	72.07	22:1n-17	117	399	576
**3**	77.50	23:1n-15	159	371	590
**4**	77.90	23:1n-16	145	385	590
**5**	78.67	23:1n-17	131	399	590
**6**	86.27	24:1n-15	173	371	604
**28**	87.30	24:1n-17	145	399	604

**Table 3:** Cytotoxic activity of the isolated AGE mixture against human cancer cell lines. Cisplatin was used as a positive control.

Cell Line	Cancer Type	IC_50_ (AGE), μg/mL	IC_50_ (Cisplatin), μg/mL
HL-60	promyelocytic leukemia	87.4 ± 23.9	0.7 ± 0.09
THP-1	monocytic leukemia	35.9 ± 4.4	3.31 ± 0.74
HeLa	cervix carcinoma	85.9 ± 17	1.55 ± 0.21
DLD-1	colon cancer	103.3 ± 21.9	9.24 ± 1.43
SNU C4	colon cancer	117.4 ± 33.1	4.01 ± 1.21
SK-MEL-28	melanoma	85.8 ± 4.7	0.89 ± 0.04
MDA-MB-231	breast cancer	137 ± 23.8	60.6 ± 26.4

## Data Availability

The original data are available from the correspondent authors on request.

## Supplementary Materials

The following supporting information can be downloaded at: www.mdpi.com/article/10.3390/md20070409/s1, Figure S1: 1H NMR spectrum of AGE mixture in CDCl3 (300 MHz); Figure S2: 13C NMR spectrum of AGE mixture in CDCl3 (75 MHz); Figure S3: DEPT spectrum of AGE mixture in CDCl3; Figure S4: GC data for the TMS-derivatives of AGEs; Figure S5: GC/MS data for the TMS-derivatives of AGEs; Figure S6: GC data for the DMDS-derivatized AGEs; Figure S7: GC/MS data for the DMDS-derivatized AGEs.
