# Supplementary material for: 1-O-Alkylglycerol Ethers from the Marine Sponge Guitarra abbotti and Their Cytotoxic Activity
Source: Mar Drugs. 2022 Jun 22;20(7):409. doi: 10.3390/md20070409 (PMC9319591; doi:10.3390/md20070409)

Supporting Information

1-*O*-Alkylglycerol Ethers from the Marine Sponge *Guitarra abbotti* and their cytotoxic activity

Sergey A. Dyshlovoy [1,2,3,†,*], Sergey N. Fedorov [4,†,*], Vasily I. Svetashev [5], Tatiana N. Makarieva [4], Anatoliy I. Kalinovsky [4], Olga P. Moiseenko [4], Vladimir B. Krasokhin [4], Larisa K. Shubina [4], Alla G. Guzii [4], Gunhild von Amsberg [1,2], and Valentin A. Stonik [4]

1 Department of Oncology, Hematology and Bone Marrow Transplantation with Section Pneumology, Hubertus Wald Tumorzentrum – University Cancer Center Hamburg (UCCH), University Medical Center Hamburg-Eppendorf, 20251 Hamburg, Germany; s.dyshlovoy@uke.de (S.A.D.); g.von-amsberg@uke.de (G.v.A.)
2 Martini-Klinik, Prostate Cancer Center, University Hospital Hamburg-Eppendorf, 20251 Hamburg, Germany
3 Institute of Science-Intensive Technologies and Advanced Materials, Far Eastern Federal University, 690091 Vladivostok, Russian Federation
4 G.B. Elyakov Pacific Institute of Bioorganic Chemistry, Far-East Branch, Russian Academy of Sciences, 690022 Vladivostok, Russian Federation; fedorov@piboc.dvo.ru (S.N.F.); makarieva@piboc.dvo.ru (T.N.M.); kaaniw@piboc.dvo.ru (A.I.K.); olga-moiseenko@yandex.ru (O.P.M.); shubina@piboc.dvo.ru (L.K.S.); gagry@rambler.ru (A.G.G.); stonik@piboc.dvo.ru (V.A.S.)
5 National Scientific Center of Marine Biology, Far-East Branch, Russian Academy of Sciences, 690041, Vladivostok, Russian Federation; vsvetashev@mail.ru (V.I.S.)
† These authors contributed equally to the manuscript.
* Correspondence: s.dyshlovoy@uke.de (S.A.D.); fedorov@piboc.dvo.ru (S.N.F.)

Figure S1. ^{1}H NMR spectrum of AGE mixture in $CDCl_3$ (300 MHz).

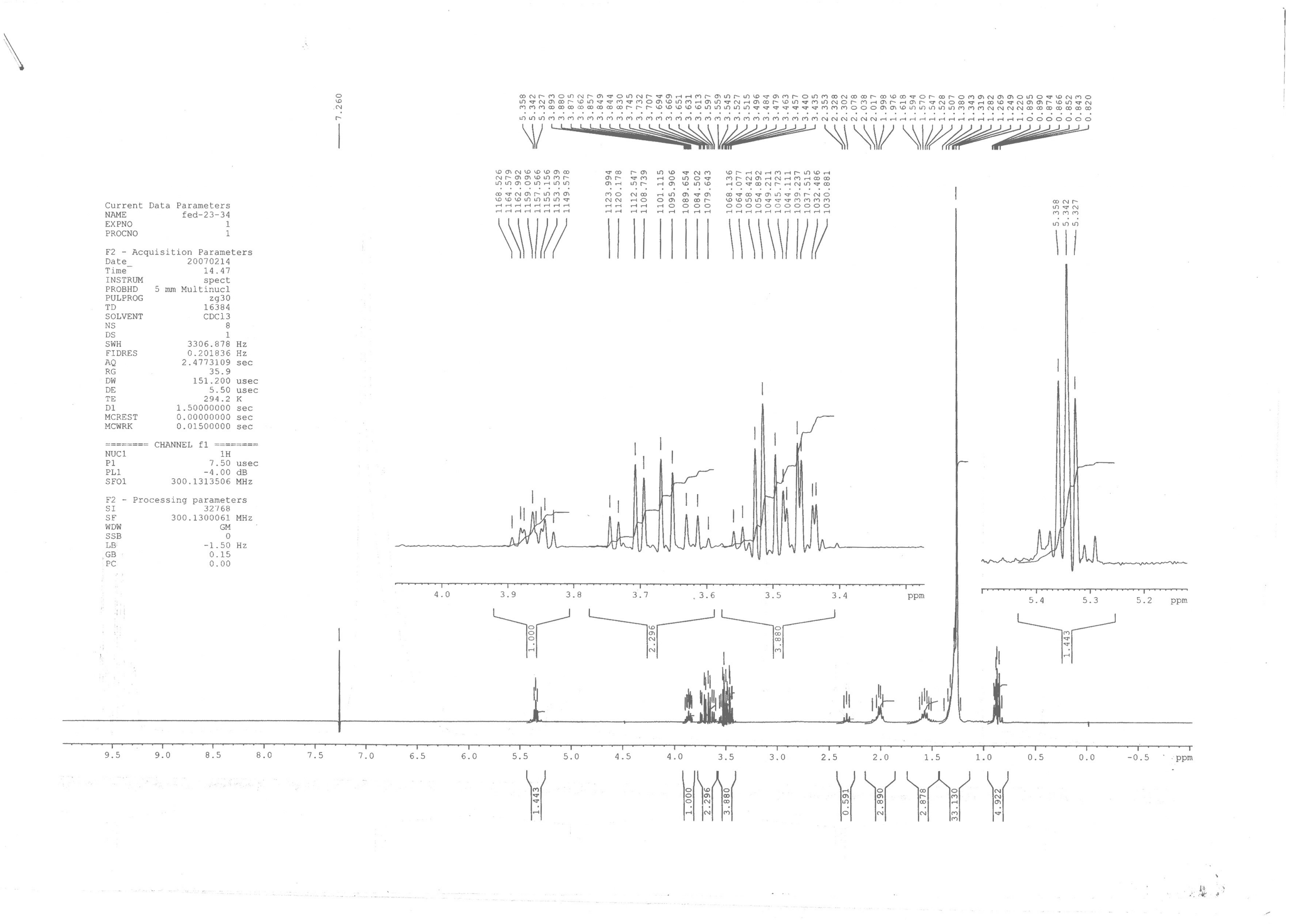

Figure S2. ^{13}C NMR spectrum of AGE mixture in $CDCl_3$ (75 MHz).

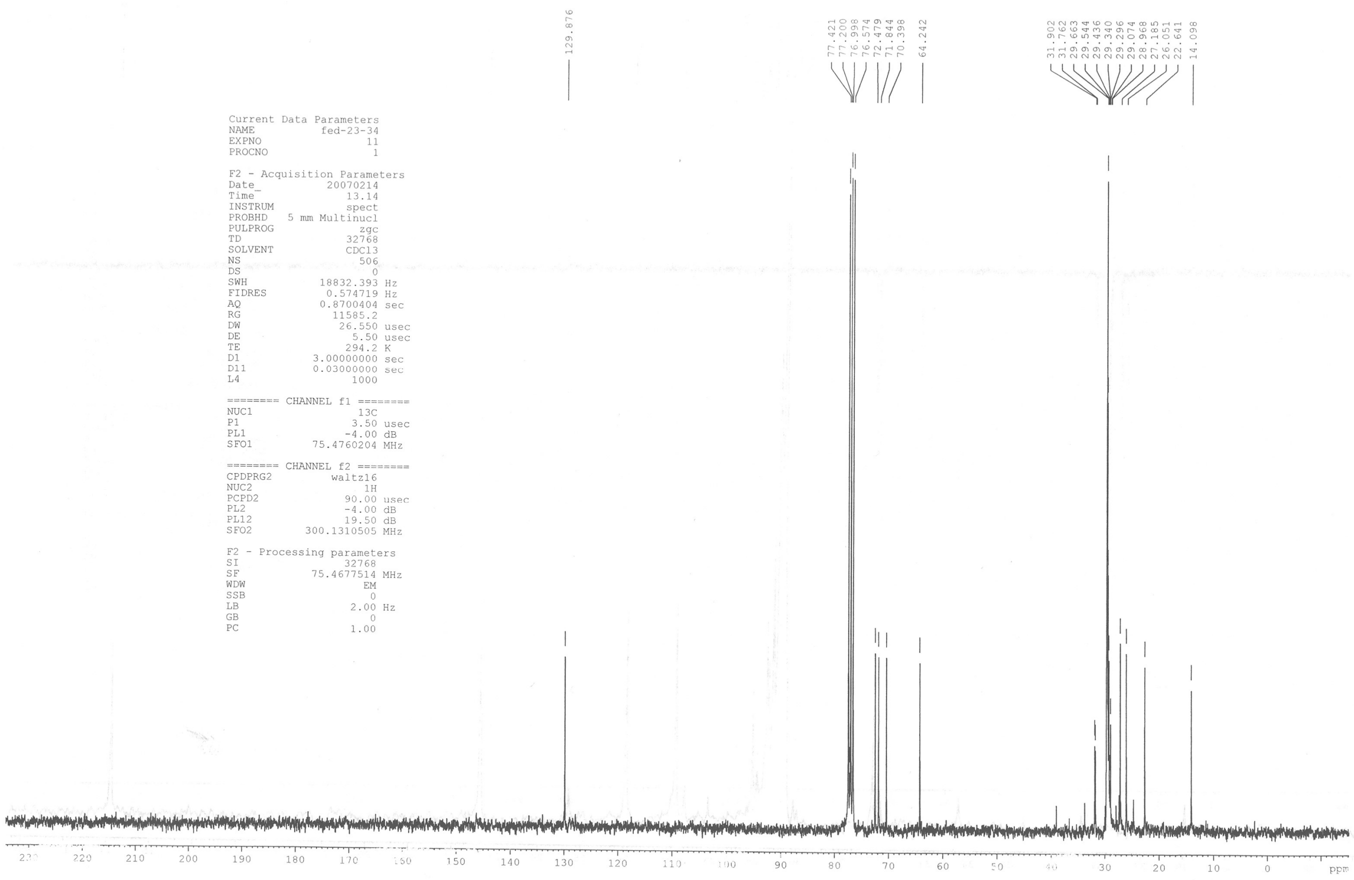

Figure S3. DEPT spectrum of AGE mixture in $CDCl_3$.

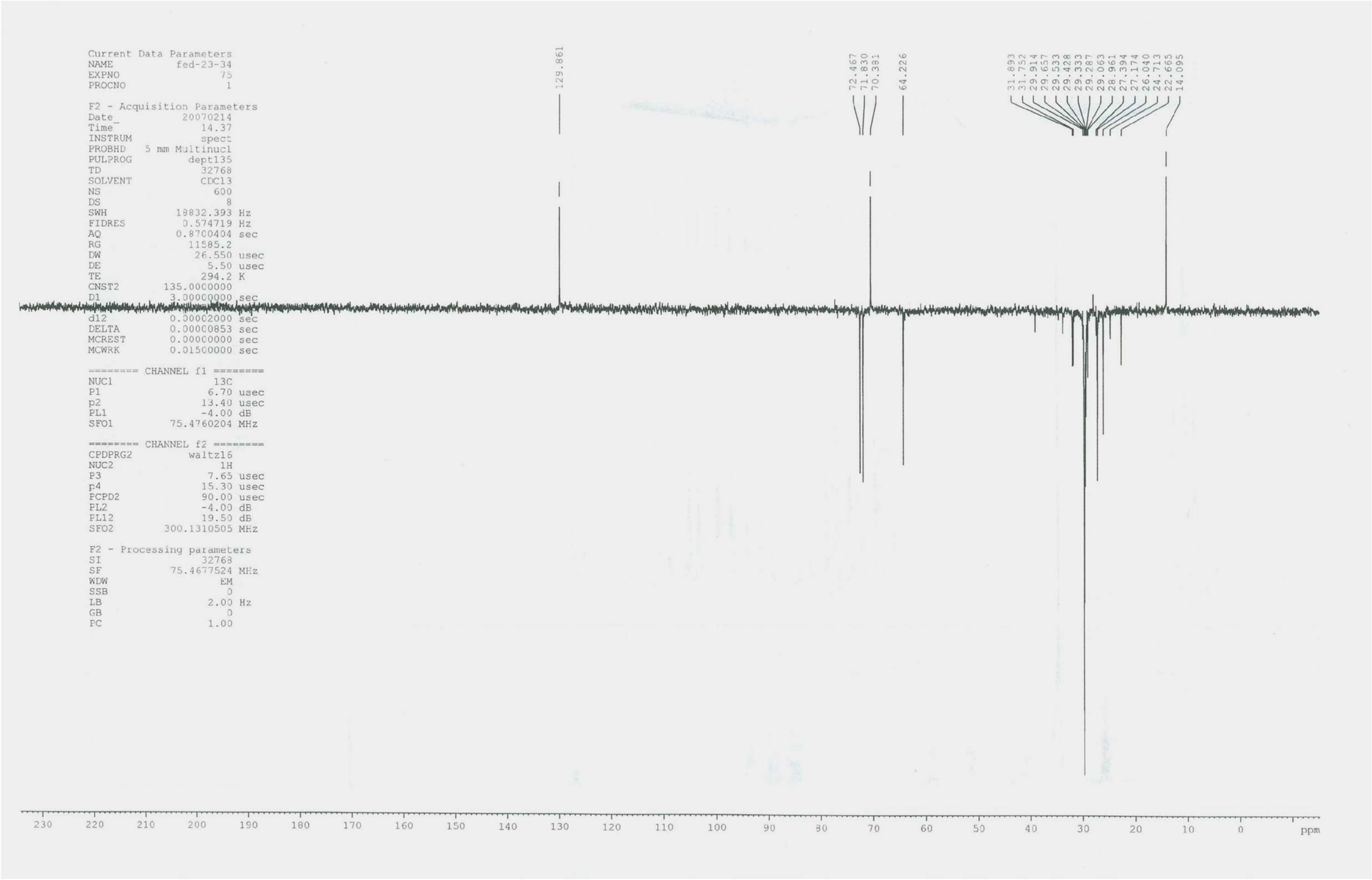

Figure S4. GC data for the TMS-derivatives of AGEs

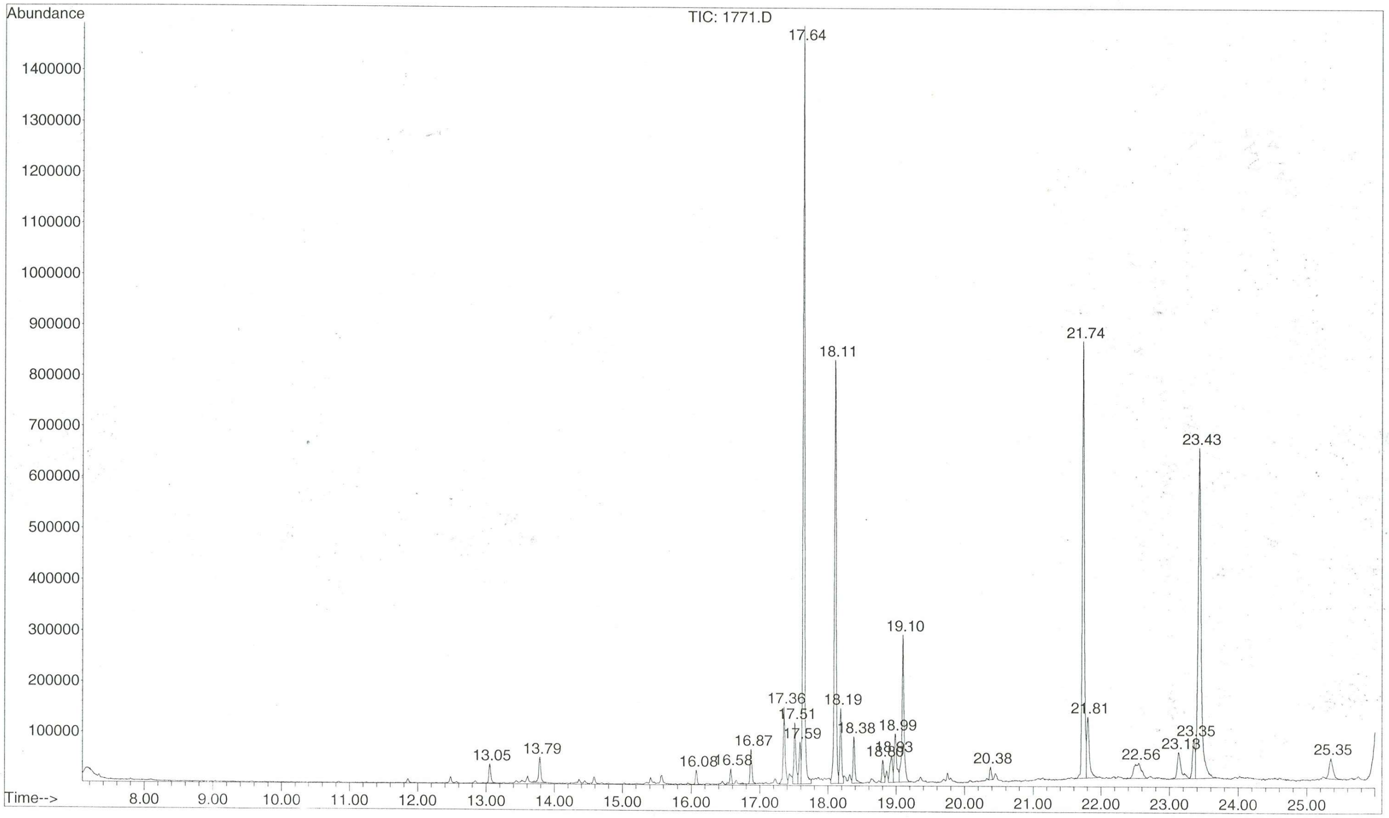

The injector and transfer line temperature was 270°C. A capillary column Hewlett Packard HP-5MS (Agilent Technologies Inc, Santa Clara, CA, USA), 30 m × 0.25 mm, phase layer 0.25 μm was used at 100°C with a 2°C/min ramp to 270°C, which was held for 30 min. Column contains 5% phenylmethylsiloxane and He was used as mobile phases, flow rate 1 mL/min. The sample was dissolved in chloroform at a concentration of 10 mg/ml. Injection volume 0.2 μL, split ratio 15:1. The mass-spectra were recorded at 70 eV.

Figure S5. GC/MS data for the TMS-derivatives of AGEs

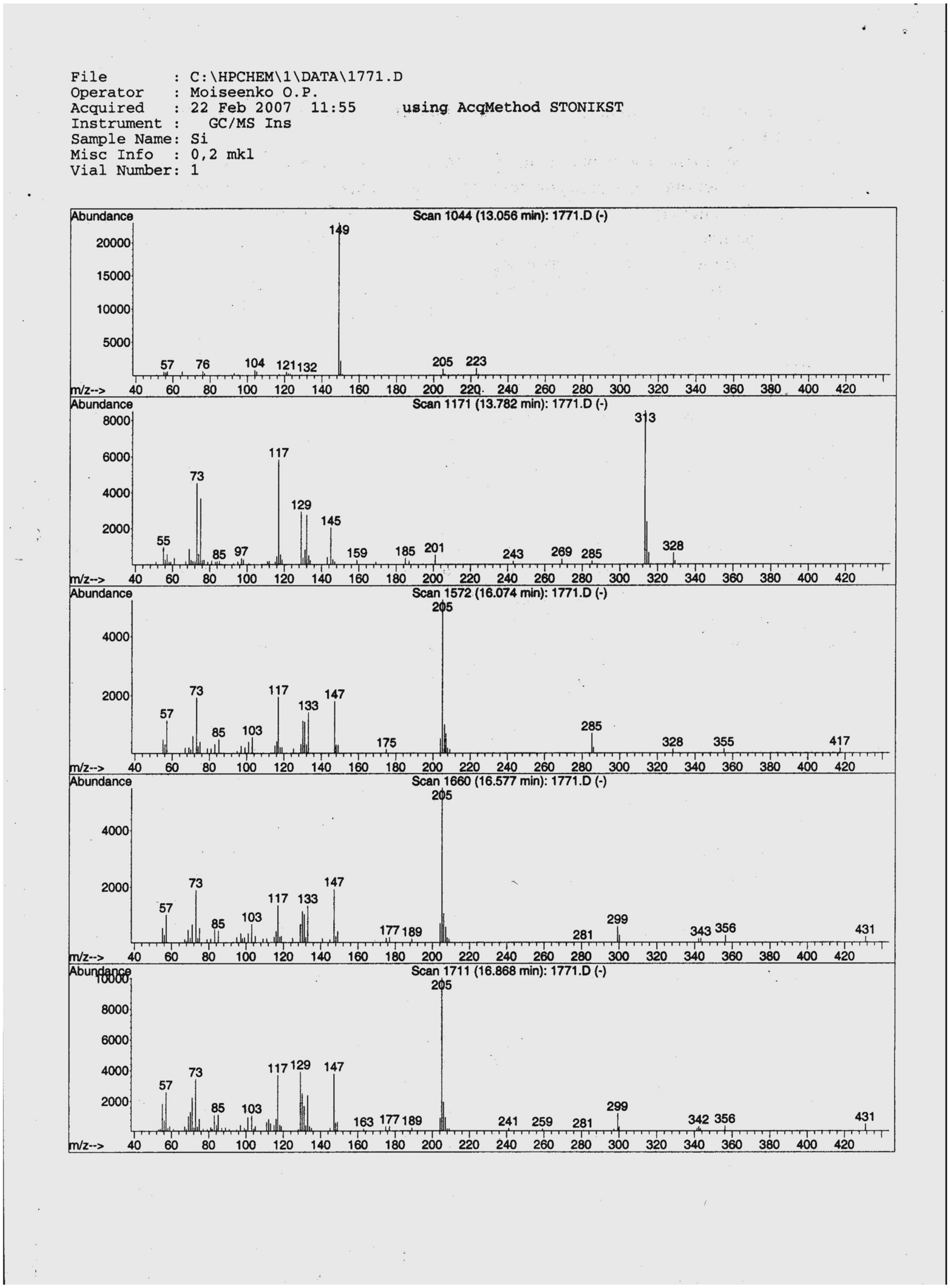

```
File       : C:\HPCHEM\1\DATA\1771.D
Operator   : Moiseenko O.P.
Acquired   : 22 Feb 2007  11:55     using AcqMethod STONIKST
Instrument :   GC/MS Ins
Sample Name: Si
Misc Info  : 0,2 mkl
Vial Number: 1
```

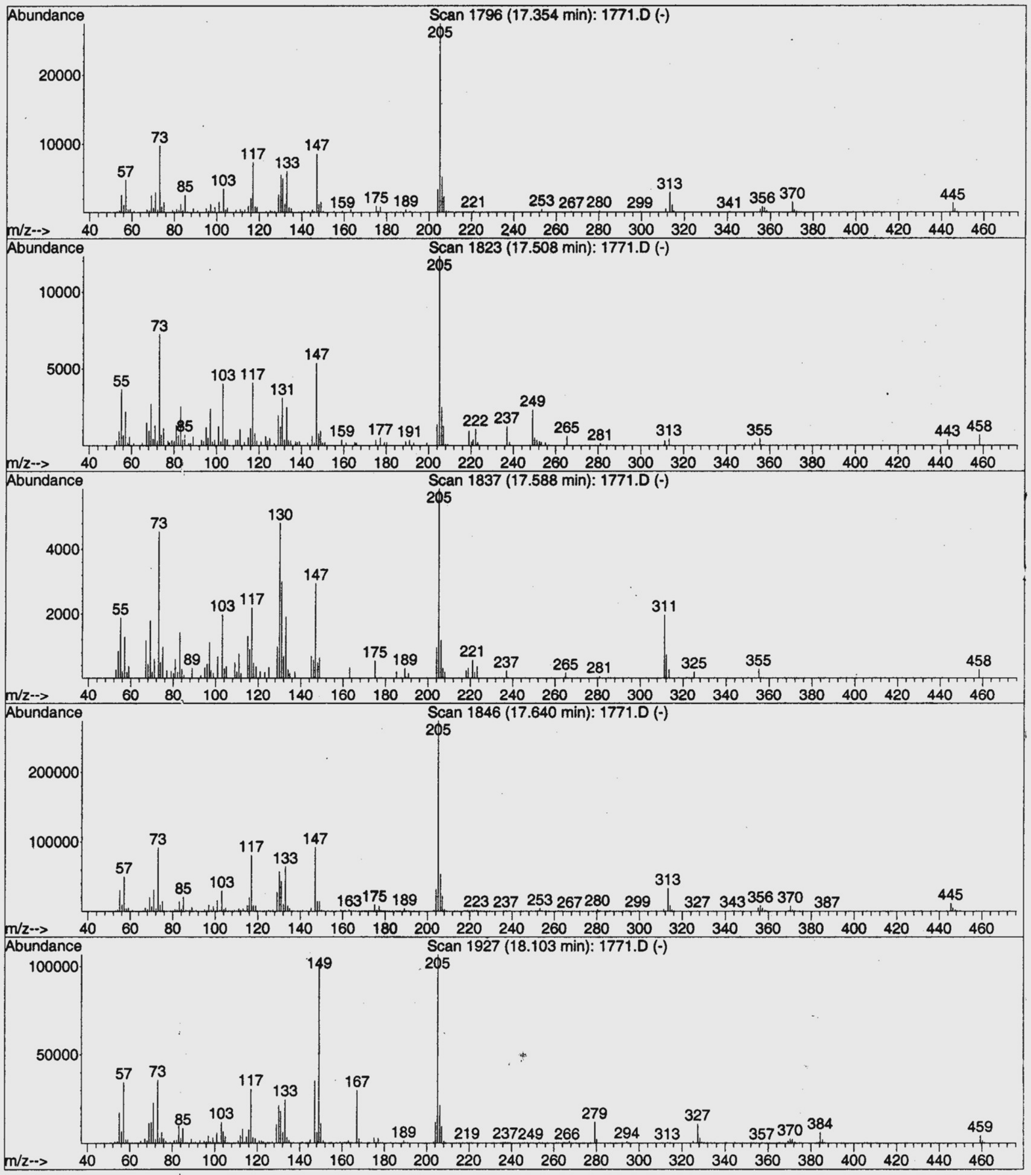

```
File       : C:\HPCHEM\1\DATA\1771.D
Operator   : Moiseenko O.P.
Acquired   : 22 Feb 2007  11:55     using AcqMethod STONIKST
Instrument :   GC/MS Ins
Sample Name: Si
Misc Info  : 0,2 mkl
Vial Number: 1
```

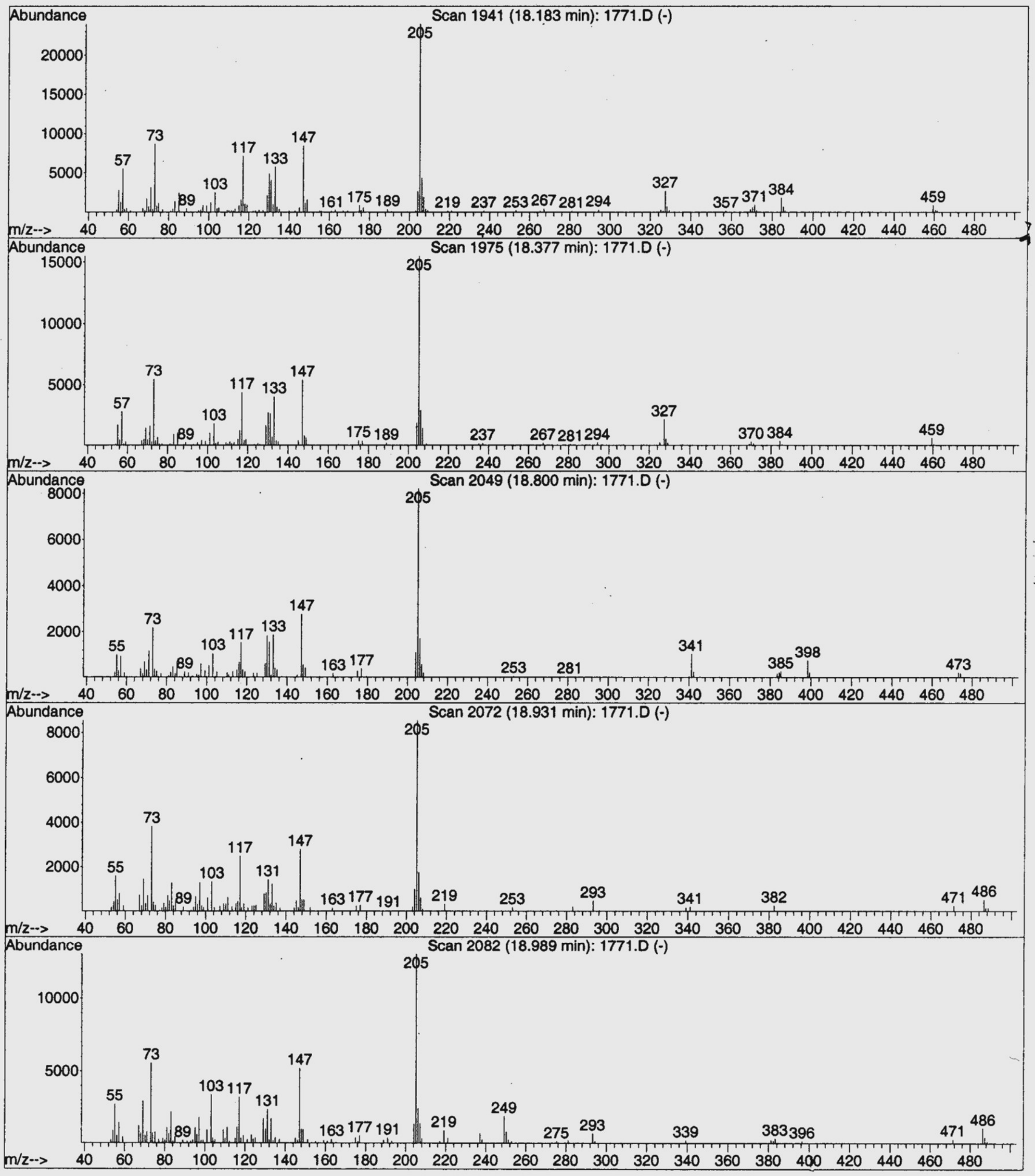

```
File       : C:\HPCHEM\1\DATA\1771.D
Operator   : Moiseenko O.P.
Acquired   : 22 Feb 2007  11:55     using AcqMethod STONIKST
Instrument :   GC/MS Ins
Sample Name: Si
Misc Info  : 0,2 mkl
Vial Number: 1
```

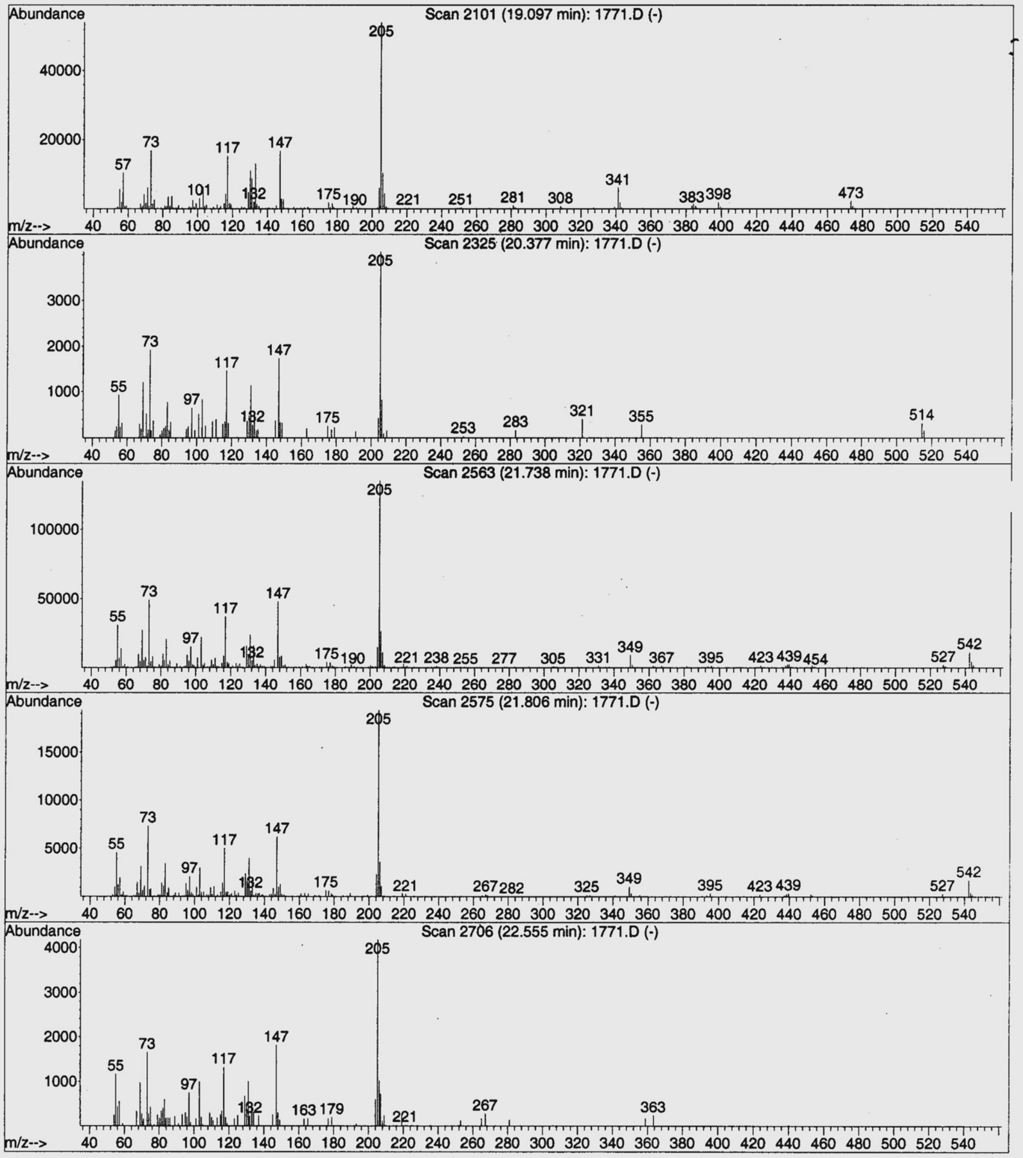

```
File        : C:\HPCHEM\1\DATA\1771.D
Operator    : Moiseenko O.P.
Acquired    : 22 Feb 2007  11:55      using AcqMethod STONIKST
Instrument  :    GC/MS Ins
Sample Name: Si
Misc Info   : 0,2 mkl
Vial Number: 1
```

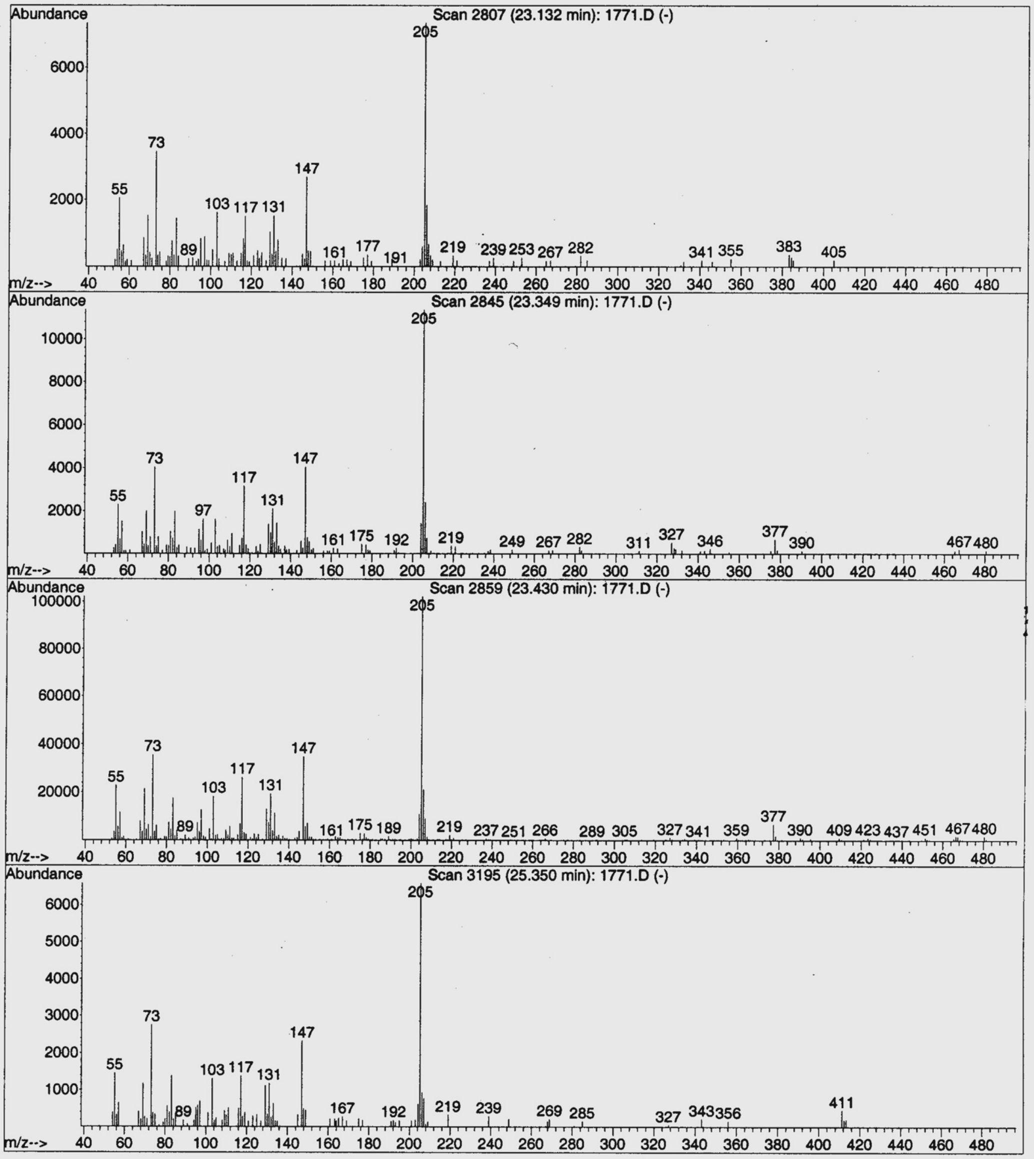

Figure S6. GC data for the DMDS-derivatized AGEs

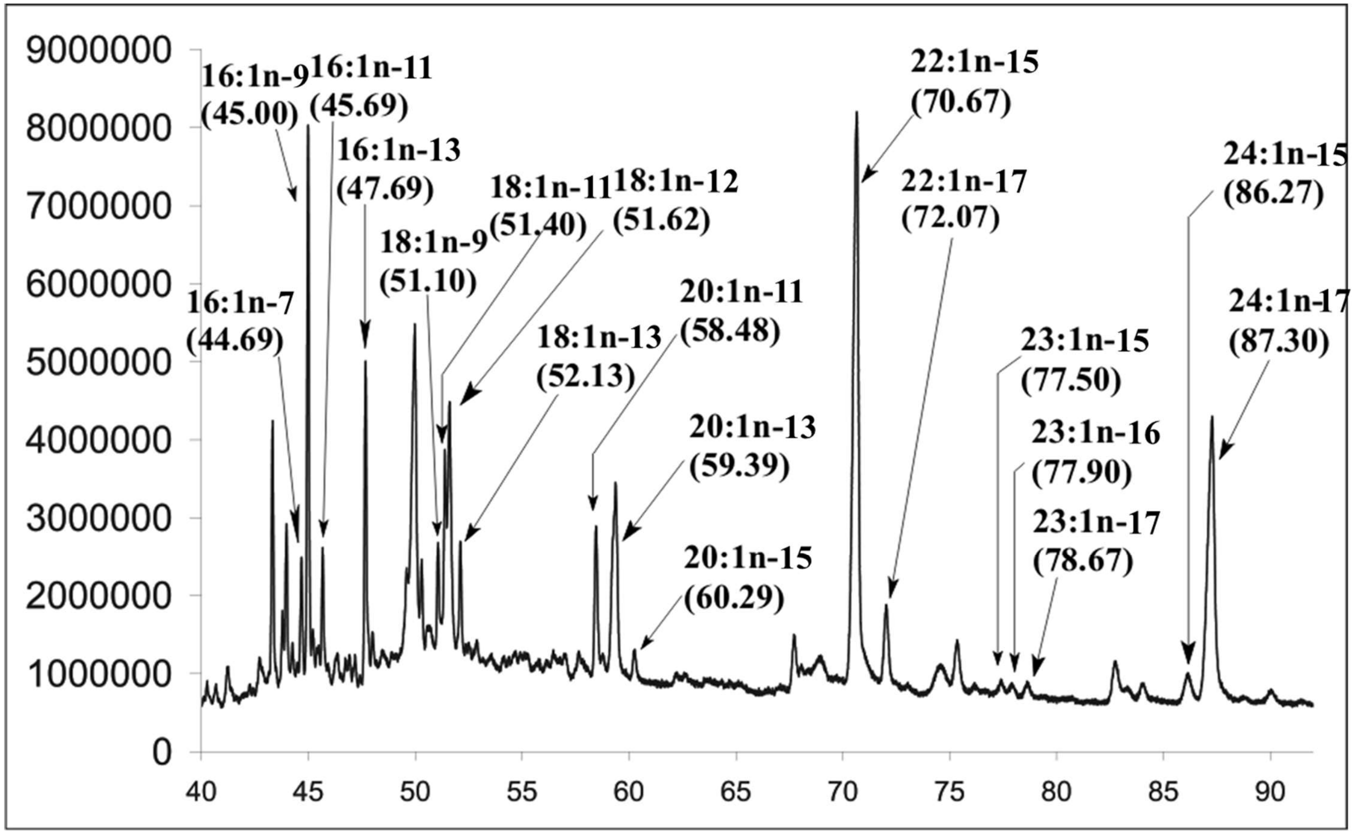

A capillary column MDN-5S (Shimadzu, Kyoto, Japan), 30 m × 0.25 mm, phase layer 0.25 μm, was used at 200 °C with a 2 °C/min ramp to 300 °C, which was held for 45 min. Split ratio 15 : 1, flow rate 1 mL/min. The injector temperature was 270 °C. The mass-spectra were recorded at 70 eV.

Figure S7. GC/MS data for the DMDS-derivatized AGEs

Compound **28** (24:1n-17, RT=87.30 min)

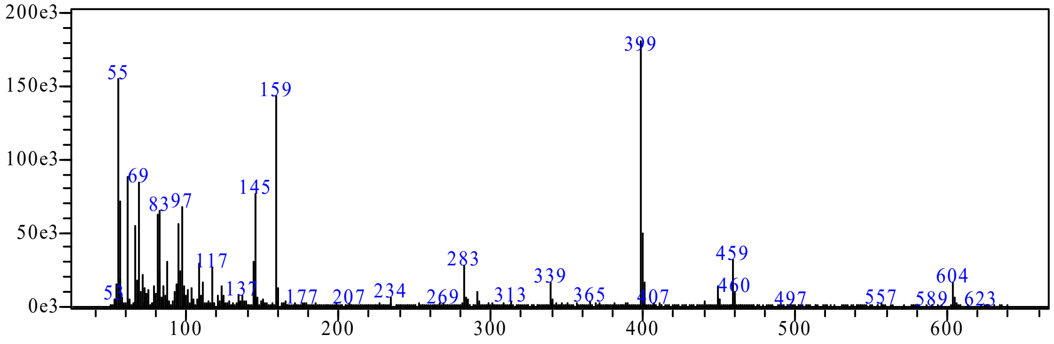

Compound **6** (24:1n-15, RT=86.27 min)

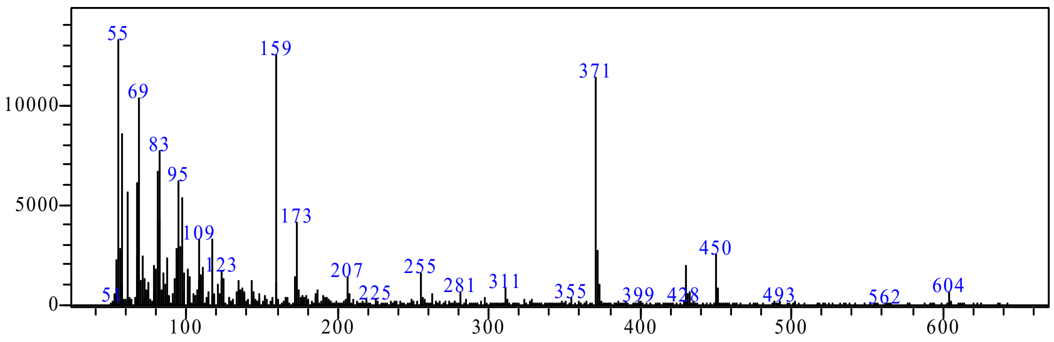

Compound **5** (23:1n-17, RT=78.67 min)

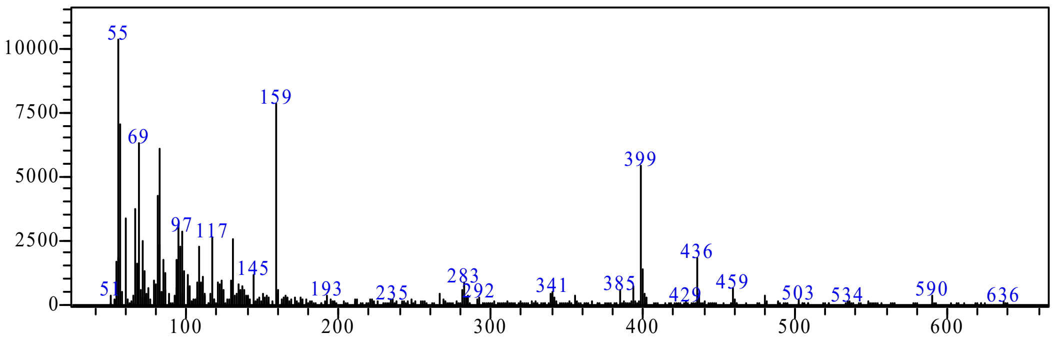

Compound **4** (23:1n-16, RT=77.90 min)

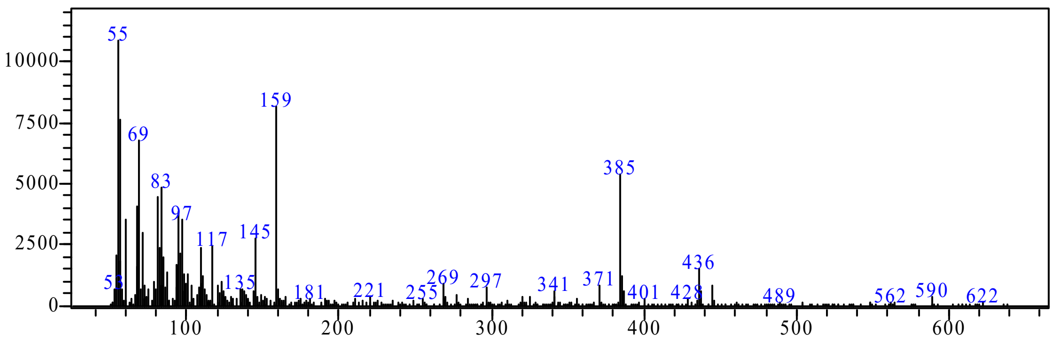

Compound **3** (23:1n-15, RT=77.50 min)

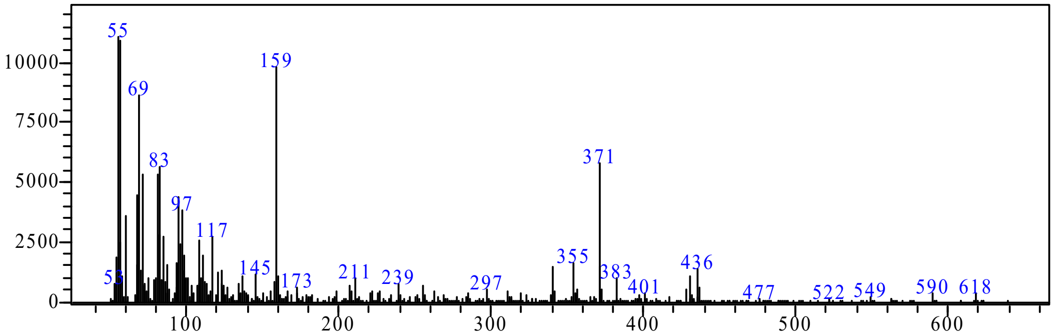

Compound **2** (22:1n-17, RT=72.07 min)

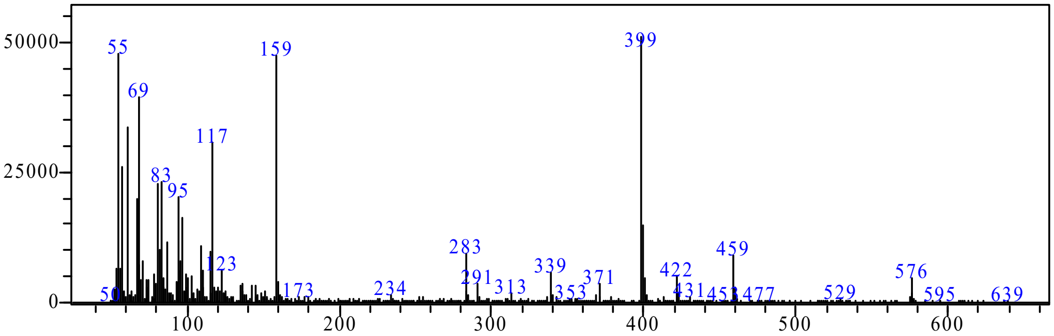

Compound **1** (22:1n-15, RT=70.67 min)

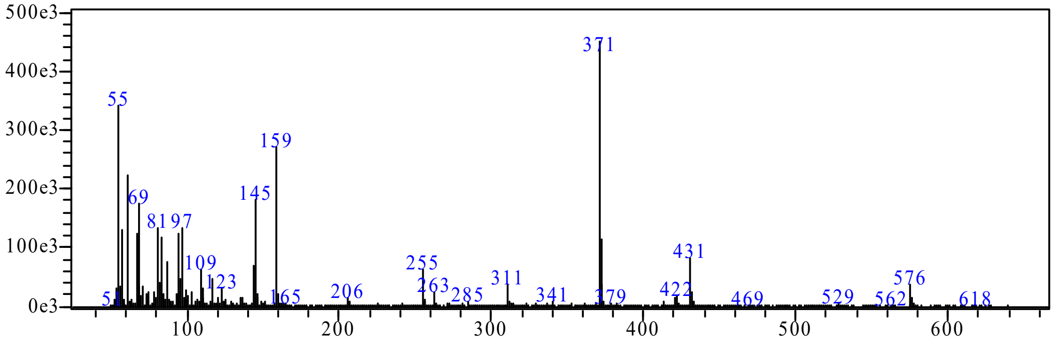

Compound **27** (20:1n-15, RT=60.29 min)

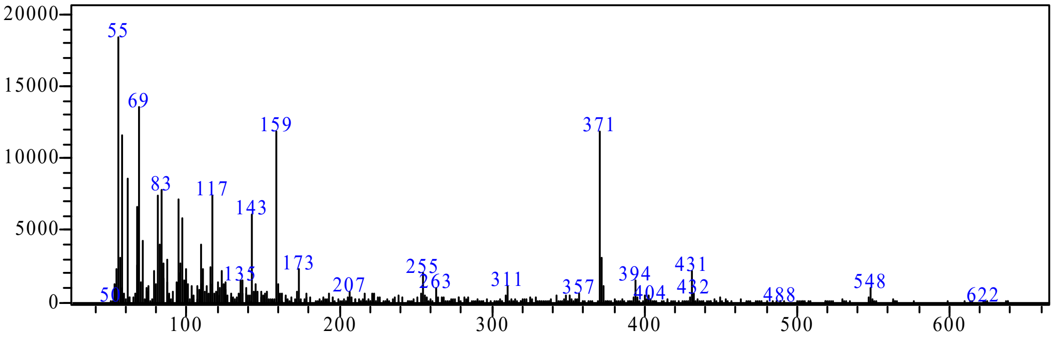

Compound **26** (20:1n-13, RT=59.39 min)

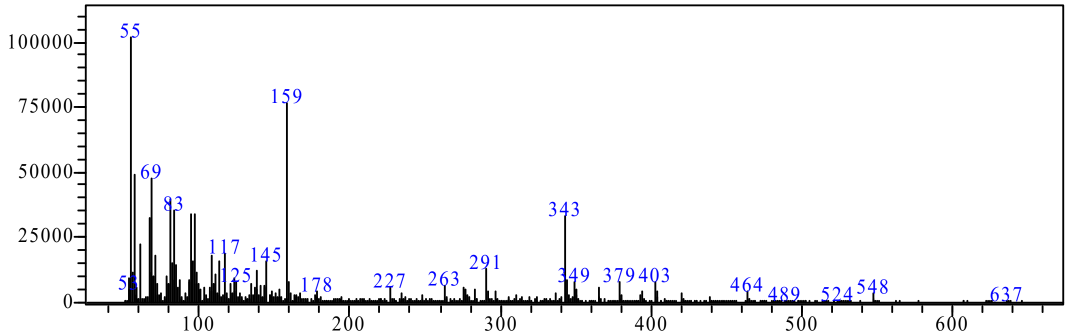

Compound **25** (20:1n-11, RT=58.48 min)

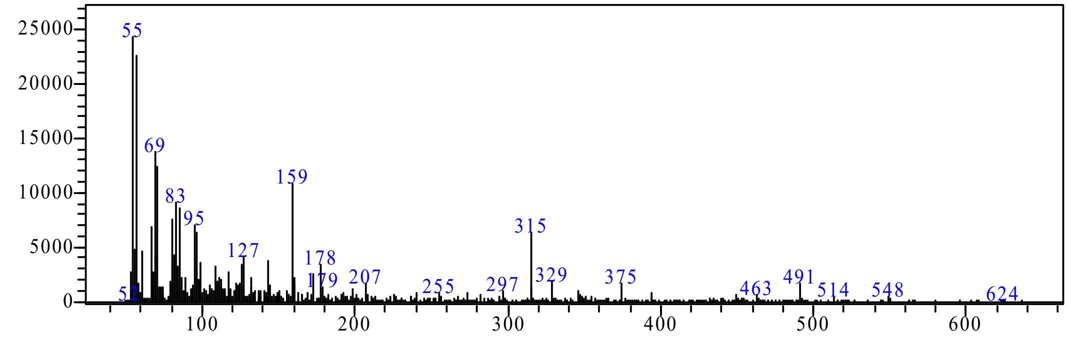

Compound **24** (18:1n-13, RT=52.13 min)

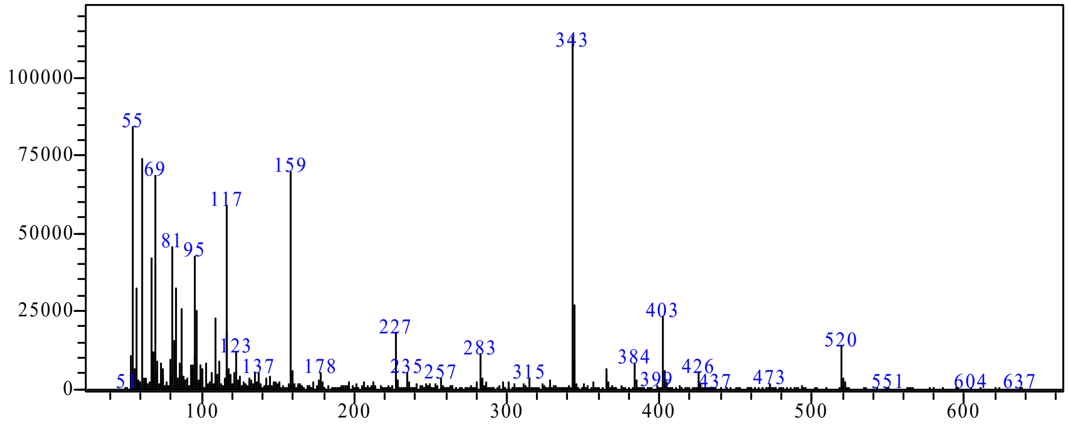

Compound **23** (18:1n-12, RT=51.62 min)

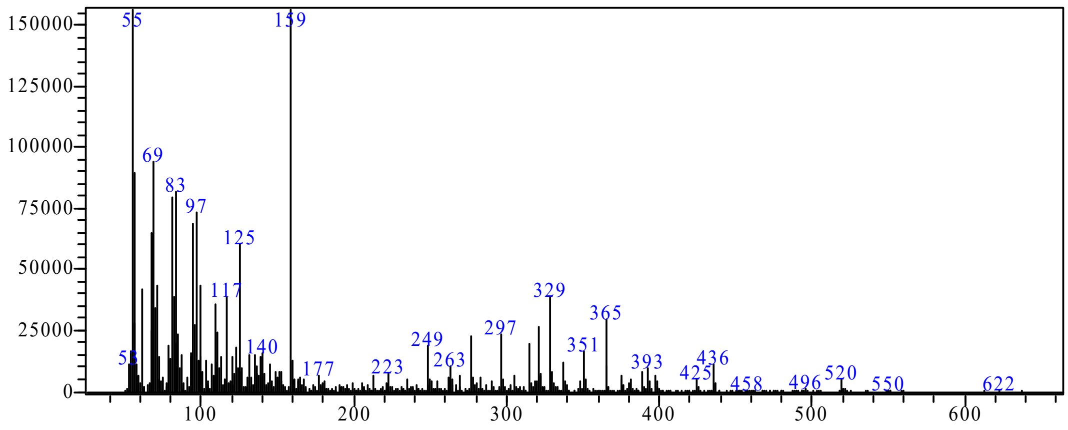

Compound **22** (18:1n-11, RT=51.40 min)

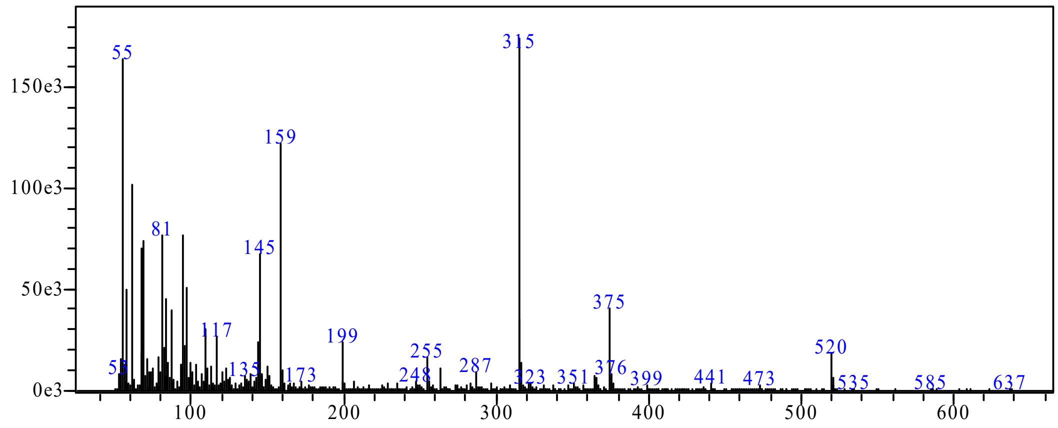

Compound **21** (18:1n-9, RT=51.10 min)

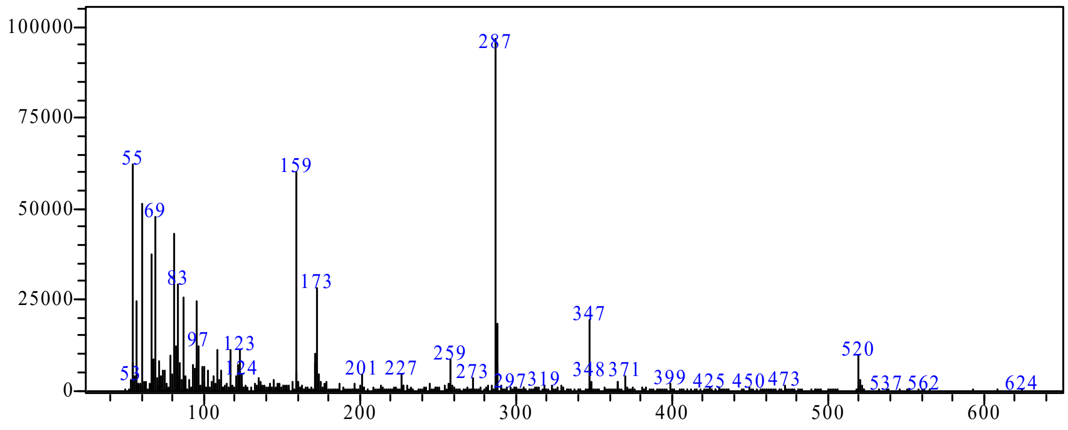

Compound **20** (16:1n-13, RT=47.69 min)

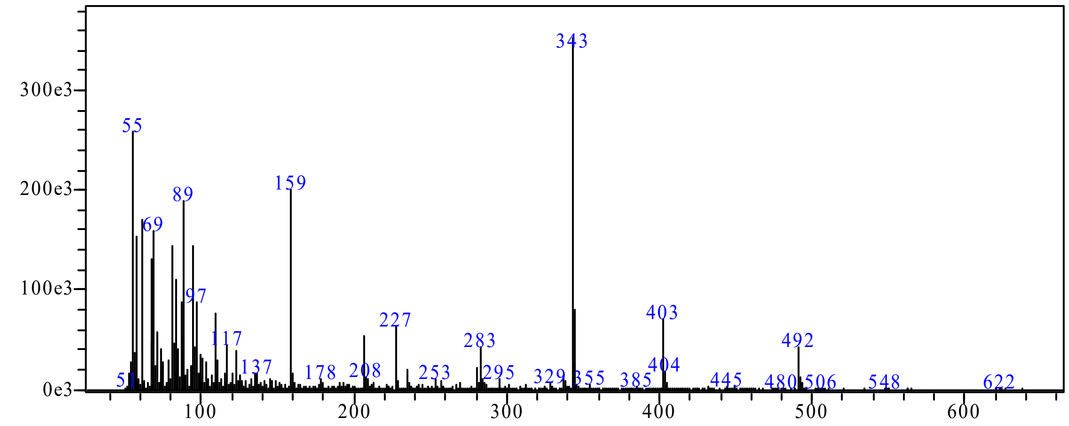

Compound **19** (16:1n-11, RT=45.69 min)

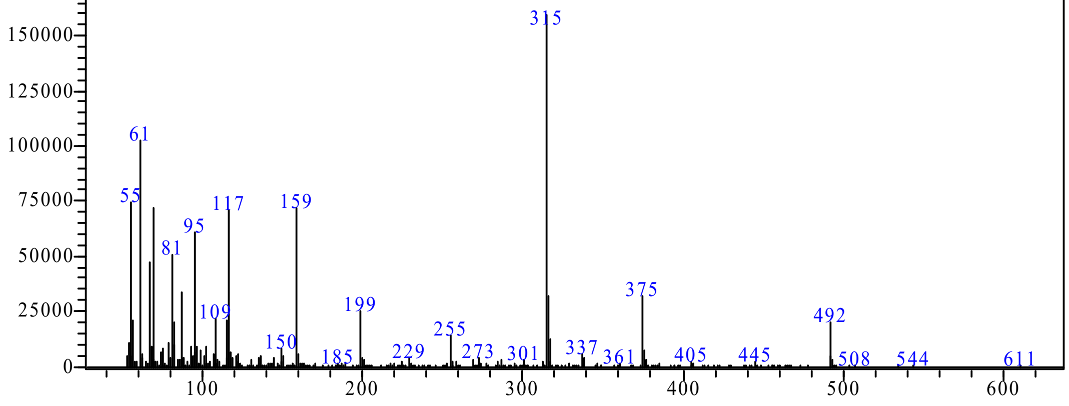

Compound **18** (16:1n-9, RT=45.00 min)

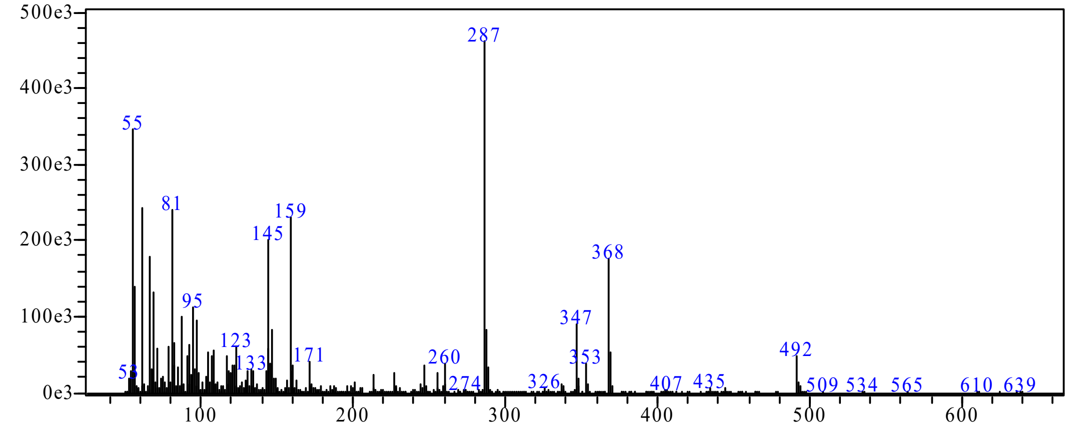

Compound **17** (16:1n-7, RT=44.69 min)

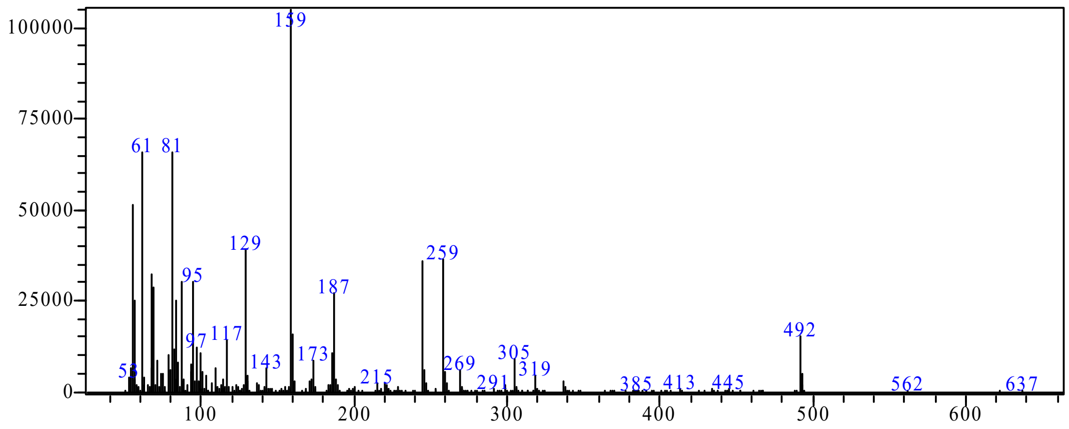

Supplement: Supplementary file 1 [file marinedrugs-20-00409-s001.zip › marinedrugs-1778628-supplementary.pdf]
